# Decomposing the True Score Variance in Rated Responses to Divergent Thinking-Tasks for Assessing Creativity: A Multitrait–Multimethod Analysis

**DOI:** 10.3390/jintelligence12100095

**Published:** 2024-09-27

**Authors:** David Jendryczko

**Affiliations:** Department for Methods for Intensive Data in Psychology, University of Konstanz, 78464 Konstanz, Germany; david.jendryczko@uni-konstanz.de

**Keywords:** alternate uses task, confirmatory factor analysis, creativity, cross-classified data, CTC (M − 1), divergent thinking, multitrait–multimethod

## Abstract

It is shown how the Correlated Traits Correlated Methods Minus One (CTC(M − 1)) Multitrait-Multimethod model for cross-classified data can be modified and applied to divergent thinking (DT)-task responses scored for miscellaneous aspects of creative quality by several raters. In contrast to previous Confirmatory Factor Analysis approaches to analyzing DT-tasks, this model explicitly takes the cross-classified data structure resulting from the employment of raters into account and decomposes the true score variance into target-specific, DT-task object-specific, rater-specific, and rater–target interaction-specific components. This enables the computation of meaningful measurement error-free relative variance-parameters such as trait-consistency, object–method specificity, rater specificity, rater–target interaction specificity, and model-implied intra-class correlations. In the empirical application with alternate uses tasks as DT-measures, the model is estimated using Bayesian statistics. The results are compared to the results yielded with a simplified version of the model, once estimated with Bayesian statistics and once estimated with the maximum likelihood method. The results show high trait-correlations and low consistency across DT-measures which indicates more heterogeneity across the DT-measurement instruments than across different creativity aspects. Substantive deliberations and further modifications, extensions, useful applications, and limitations of the model are discussed.

## 1. Introduction

Divergent thinking (DT) is arguably the best psychological construct for approximating a quantitative display of inter-individual differences in human creativity in a psychometric and test-theoretical manner and is useful for predicting creative achievement (Guilford 1966; Lubart et al. 2010; Kim 2008; Runco and Acar 2012; Wallach and Wing 1969). It is understood as a person’s ability to produce several ideas or solutions to a given problem or task (Guilford 1967). One example for a concrete DT-task is the insight test or, alternatively labeled, the finding explanations task (FET; Forthmann et al. 2019; Jäger et al. 2006; Preckel et al. 2011): A subject has to come up with explanations for a certain circumstance. For example: Why do many people think of person X as choleric? One explanation might state that X scolds her or his employees all the time for only minor mistakes; another explanation might state that X yells at potatoes for not peeling themselves. Most people would probably deem the second explanation more creative due to its humorous absurdity alone. 

Another established method for assessing DT is the alternate uses task (AUT; Guilford 1967; Wallach and Kogan 1965). In such a task, a subject has to write down as many alternate uses for an everyday object like a kitchen knife, for example. “Alternate” means that the use should be different to the “normal” use of the object. For a kitchen knife, the normal use would be to cut food; examples for alternate uses might be to use the reflective surface of the blade as a mirror or to stick the knife into a wall so that its handle can be used as a coat hook.

The derivation of a quantitative DT-score is anything but straightforward. Deliberations must be made even before idea production, as DT-tasks can come with different instructions for the target subjects. The instruction can be to produce as many ideas as possible (“be-fluent”; e.g., Runco and Acar 2010) or to focus on the production of creative and unusual ideas (“be-creative”; e.g., Nusbaum et al. 2014) with the latter—at least in principle—better reflecting the concept of creativity. After ideas have been gathered, one may simply use the number of produced ideas (fluency) as an indicator for DT or consult sample-independent raters to judge the creativity of the produced ideas (see, for example, Forthmann et al. 2017). The latter process, again, lends itself more towards the concept of creativity and can, in turn, be sub-divided into several more specific scoring procedures. Raters may judge every single idea of a target or give so called “snapshot-ratings”, i.e., rate the overall set of ideas of each target (e.g., Silvia et al. 2009). Moreover, they may rate the (set of) ideas with regards to different aspects like uncommonness, remoteness (e.g., for an AUT: behavioral distance of the alternate use to the common use; using a knife as a mirror is arguably more remote than using it as a coat hook as the latter use still implies the process of cutting when the knife is inserted into the wall), cleverness, which encompasses imaginativeness, ingenuity, funniness, and cunning aptness (French et al. 1963; Johnson et al. 1968; Mullins 1963; Stratton and Brown 1972; Wilson et al. 1953), and usefulness (Runco and Jaeger 2012; probably more appropriate for AUTs than for FETs). When raters are instructed to include all or a subset of the aforementioned aspects in their ratings, the ratings are usually stated to indicate (overall) creative quality (e.g., Forthmann et al. 2017). Note that the employment of (at least two) raters implies a cross-classified data structure for DT scores (given that all raters rate all targets). Variability in ratings is potentially attributable to differences in targets, differences in raters (independent higher levels), and differences in rater–target dyads/interactions (lower interaction level; see, for example, Koch et al. 2016).

**Figure 1:**
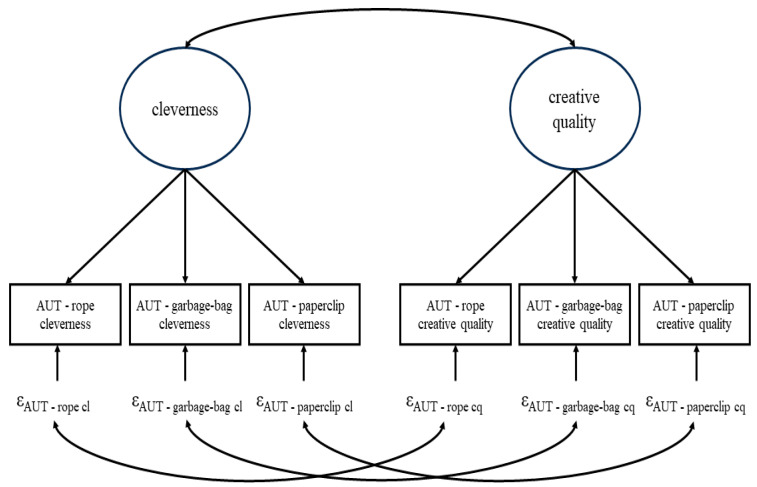
Example for the standard CFA approach to analyzing divergent thinking (DT). In this example, three different AUTs each scored both for cleverness and creative quality were used. ε depicts a residual. cl = cleverness, cq = creative quality.

On top of this plethora of different instructions and scoring procedures, often, several DT-tasks are given to targets instead of only one. For example, targets might answer three AUTs with the respective objects being a rope, a garbage bag, and a paperclip (Forthmann et al. 2017). This is useful from a psychometric perspective as several tasks that are intended to measure the same construct can be used to separate the true score from measurement error within the framework of classical test theory using confirmatory factor analysis (CFA; Bollen 1989). Once the ratings of all raters for all targets on all DT-tasks are collected, the data is often analyzed in the following way (e.g., Forthmann et al. 2017, 2019): for each DT-task or the mean across all DT-tasks, an intra-class correlation coefficient (ICC) is computed. The ICC displays the proportion of variance in the ratings that can be attributed to the targets. Therefore, the higher the value (that can range from 0 to 1) the more consent among the raters is present (usually, a minimum value of 0.7 is aimed at). Note that by using the raw ratings, measurement error is not partialized out before the ICC is computed. Afterwards, some sort of aggregate among raters is computed for each DT-task and target, so that one value is given for each target on every DT-task. With those values, a standard CFA is estimated. If multiple rating-procedures were applied—say cleverness ratings and separate creative quality (encapsulating uncommonness and remoteness) ratings for the three AUTs as mentioned above (see Figure 1)—one target-specific score variable is computed for every combination of DT-task and rating procedure. The DT-scores relating to the same aspect/rating procedure (e.g., cleverness ratings) are combined to measure the same latent variable (e.g., latent cleverness); the latent variables and their covariances represent the overarching DT-construct. Partial covariances of residuals relating to the same DT-tasks but different scoring procedures are modeled to respect possible DT-object dependencies. With “DT-object”, we refer to the concrete available information within a DT-task. This is literally a material item for which alternate uses are sought for in AUTs but more abstract in other DT-tasks, e.g., the choleric personality in the FET-example above. Note that measurement error variance and DT-object specific variance are confounded in the residual variance within this simple CFA approach.

The purpose of the current contribution is to present an alternative CFA approach to analyzing rated responses to DT-tasks. By conceptualizing different creativity aspects (like cleverness and creative quality) as different traits, different DT-objects as structurally different methods and different raters as interchangeable methods, DT-scores can be analyzed with Multitrait–Multimethod (MTMM; Campbell and Fiske 1959) factor models. The particular MTMM–CFA approach utilized in the current contribution is a modification of the Correlated Traits Correlated Methods Minus One (CTC(M − 1); see (Eid 2000; Eid et al. 2003, 2008; Nussbeck et al. 2006, 2009) model for cross-classified data (C4 for short; Koch et al. 2016). The model includes all variance components (targets, raters, rater–target interactions, DT-task objects, and measurement error) by fully respecting the cross-classified data structure of rated DT-responses in the model itself, directly adheres to the stochastic sampling process given in cross-classified DT scoring-procedures, and is formally definable in the framework of classical test theory and stochastic measurement theory (Steyer 1989; Zimmerman 1975). The following advantages over the standard CFA approach as depicted in Figure 1 are gained:

1. Model-implied ICCs can be computed for the DT-scores within a specific rating procedure (construct) that only consider variability of true scores and separate measurement error;
2. DT-object-specific variability can be separated from measurement error;
3. The model allows for the computation of additional informative relative true-score variance components such as various forms of consistency and method specificity;
4. Using Bayesian methods, credibility intervals (CRIs) for all relative variances (mentioned in 1. and 3.) can be computed;
5. Rater-effects (variability across raters) can be separated from interaction-effects (variability across rater–target interactions) which allows one to investigate whether raters consistently maintain their standards across targets;
6. Due to the flexibility of SEM, the model can be extended to include attributes of raters in order to predict differences in raters, for example (the same is true for rater–target interactions).

In the following, we formally define the model in the frameworks of classical test theory. We display the variance decomposition and introduce various types of relative variances that can be computed. Lastly, we apply the model (and a simplification of it) to data on cleverness and creative quality ratings for the three different AUT-tasks as also shown in Figure 1.

## 2. Defining an Appropriate Cross-Classified CTC(M − 1) Model for DT-Ratings

**Figure 2:**
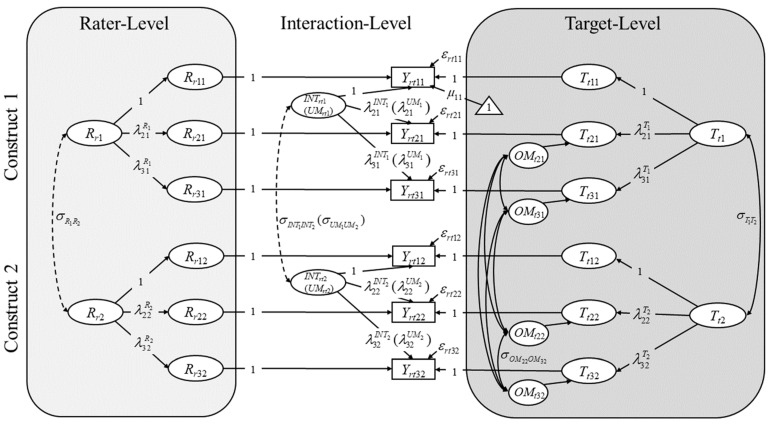
The Divergent Thinking Cross-Classified model (DTCC) as a modified version of the C4 (Koch et al. 2016) with indicator-specific method factors (e.g., Geiser and Simmons 2021). In this example, three DT-tasks (e.g., three AUTs with the objects of rope (*i* = 1), garbage bag (*i* = 2), and paperclip (*i* = 3)) and two constructs (e.g., cleverness (*j* = 1) and creative quality (*j* = 2)) are given. The first DT-indicator/object (*i* = 1) is defined as a reference method for all constructs. All factor loadings (denoted by *λ*) for this indicator are set to 1. *Y_rtij_* = observed rating of rater *r* for target *t* on DT-object (indicator) *i* for construct *j*. *ε_rtij_* = residual of an observed rating. $\mu_{ij}$ denotes an intercept/unconditional expectation for indicator *i* and construct *j* (only exemplarily depicted for the first indicator of the first construct). *T_tj_* = latent trait variable for construct *j*. $\lambda_{ij}^{T_{j}}T_{tj}$ denotes an expected deviation from the intercept as assessed with the reference method *i* = 1 given target *t*. *OM_tij_* = (DT-) object-specific (indicator-specific) method effect variable for non-reference object *i* ≠ 1 for construct *j*. It depicts the expected deviation from $\lambda_{ij}^{T_{j}}T_{tj}$ given the non-reference method *i* ≠ 1. *R_rj_* = rater effect variable for construct *j*. $\lambda_{ij}^{R_{j}}R_{rj}$ denotes an expected deviation from the intercept given rater *r*. *INT_rtj_* = interaction effect variable for construct *j*. $\lambda_{ij}^{INT_{j}}INT_{rtj}$ depicts the expected deviation from the intercept, $\lambda_{ij}^{T_{j}}T_{tj}+OM_{tij}$, and $\lambda_{ij}^{R_{j}}R_{rj}$ given the specific combination of target *t* and rater *r*. *σ* denotes a covariance. Note that all *OM_tij_* may covary with each other but only the covariance between *OM_t_*_22_ and *OM_t_*_32_ is labeled exemplarily to maintain visual clarity. Dashed double arrows indicate covariances that can only be modeled if the same raters (at least in part) are consulted for both constructs. In the simplified Divergent Thinking Two-Level model (DTTL), the interaction-level is described as level-1 and the target-level is described as level-2 (there is no rater-level) and the latent variables on level-1 are more appropriately referred to as unique method-effect variables (UMrtj; see Eid et al. 2008; Koch et al. 2016).

The original C4 (Koch et al. 2016) was derived and illustrated for cases in which self-reports (e.g., of personality traits like academic interest) of study participants/targets (e.g., students) are augmented by other reports provided by a distinct set of interchangeable raters (e.g., the teachers of the students). Other ratings reflect a structurally different method to the “gold-standard” method (Eid et al. 2003) of self-reports. A common method effect (reflecting rater-agreement or their “common perspective” with regard to a target; see also Eid et al. 2008) can be defined as a residual to the trait as assessed with the standard method of self-reports. In DT-studies, creativity self-ratings of targets are usually not given, and the different DT-objects reflect the structurally different assessment methods of interest. Therefore, the C4 can be applied with a modification: one DT-task/indicator (containing a specific DT-object) can be defined as a standard method for assessing any creativity aspect (trait) of a target using the common perspective of multiple interchangeable raters and indicator- (or object-) specific method effects (e.g., Bishop et al. 2015; Eid and Diener 2004; Eid et al. 2003; Geiser et al. 2008; Geiser and Lockhart 2012; Geiser and Simmons 2021; Koch et al. 2018; Schmitt and Steyer 1993) of the structurally different non-standard methods (indicators/objects) can be modeled while still respecting different raters as interchangeable methods. In the following paragraphs, we show how such a modified C4 model can be defined for DT-ratings within classical test theory. For convenience, we refer to this model as the Divergent Thinking Cross-Classified model (DTCC). We refer to a simplified version of this model that conglomerates rater and rater–target interaction effects as the Divergent Thinking Two-Level model (DTTL). Readers already familiar with the C4 and the modeling of indicator-specific method effects within the CTC(M − 1) tradition, and readers primarily interested in the meaning of latent variables instead of technical aspects of model definition, may refer to Figure 2 and its description for the final model(s) (see also Equations (7)–(9)) and skip ahead to the next section.

All latent variables of the DTCC are defined as conditional expectations or deviations thereof in the framework of stochastic measurement theory where targets and raters are treated as outcomes of random variables. The stochastic sampling space for cross-classified data including mapping and the definition of conditional expectations is given in Koch et al. (2016). Let $Y_{rtij}$ denote the given score of rater *r* to target *t* on DT-indicator *i* for construct *j*. For example, *i* might have three levels (*i* = 1, 2, or 3), denoting the AUTs of rope, garbage bag, and paperclip, respectively. *j* might have two levels (*j* = 1 or 2) with 1 denoting cleverness and 2 denoting creative quality. If individual idea ratings are used (as opposed to snapshot ratings) one needs to obtain $Y_{rtij}$ by computing a rater–target combination specific aggregate for DT-task *i* of construct *j* like the mean or, preferably, the 0.75 quantile of the idea ratings (see, for example, Forthmann et al. 2017). Within classical test theory, each of these ratings can be decomposed into a true score $\tau_{rtij}$ and a measurement error $\varepsilon_{rtij}$:

(1) $$Y_{rtij}=\tau_{rtij}+\varepsilon_{rtij}.$$

Next, in accordance with the CTC(M − 1) tradition, one needs to define one indicator *i* as a reference indicator/method. For example, for the three AUTs mentioned above on might set indicator *i* = 1 (rope) is the reference indicator (note that *i* denotes an indicator/method and an DT-object simultaneously). For this reference indicator, the true score can be further decomposed into an unconditional expectation (intercept) across all raters and targets $\mu_{1j}$, an expected conditional deviation from this intercept given the target (trait) $T_{t1j}$, an expected conditional deviation from this intercept given the rater (rater-effect) $R_{r1j}$, and a deviation for the interaction (combination) of rater and target (interaction-effect) $INT_{rt1j}$:

(2) $$\tau_{rt1j}=\mu_{1j}+T_{t1j}+R_{r1j}+INT_{rt1j}$$

(see Koch et al. 2016). For the true score of the remaining non-reference indicators (*i* ≠ 1) of construct *j*, the same decomposition holds:

(3) $$\tau_{rtij}=\mu_{ij}+T_{tij}+R_{rij}+INT_{rtij}.$$

We regress the target-specific latent trait variable for any non-reference indicator $T_{tij}$ on the latent trait of the reference indicator $T_{t1j}$, giving:

(4) $$T_{tij}=\lambda_{ij}^{T_{j}}T_{t1j}+OM_{tij}.$$

Here, $\lambda_{ij}^{T_{j}}T_{t1j}$ denotes an expected trait value for the non-reference DT-object *i* given the trait value for the reference DT-object *i* = 1 and $OM_{tij}$ depicts a construct (*j*) and DT-object specific residual for DT-object i—the object-specific (or indicator-specific) method effect (Bishop et al. 2015; Eid and Diener 2004; Eid et al. 2003; Geiser et al. 2008; Geiser and Lockhart 2012; Geiser and Simmons 2021; Koch et al. 2018; Schmitt and Steyer 1993).

For the $R_{rij}$ and $INT_{rtij}$ of Equations (2) and (3), we assume that they are, respectively, linear transformations of each other across different indicators (*i* ≠ *i′*): $R_{rij}=\lambda_{ii^{\prime }}^{R_{j}}R_{ri^{\prime }j}$, and $INT_{rtij}=\lambda_{ii^{\prime }}^{INT_{j}}INT_{rti^{\prime }j}$. Note that no intercepts are given in the linear equations $T_{t1j}=\lambda_{ij}^{T_{j}}T_{t1j}+OM_{tij}$, $R_{rij}=\lambda_{ii^{\prime }}^{R_{j}}R_{ri^{\prime }j}$, and $INT_{rtij}=\lambda_{ii^{\prime }}^{INT_{j}}INT_{rti^{\prime }j}$ as all of the latent variables depict deviations and, thus, have expectations of zero. We can impose, without loss of generality, a congeneric measurement structure on the $R_{rij}$ and $INT_{rtij}$, respectively, by defining the metric of latent variables for the reference indicator as the standard ($R_{r1j}=R_{rj}$ and $INT_{rt1j}=INT_{rtj}$) and define the respective latent variables of non-reference indicators as transformations thereof: $\lambda_{ij}^{R_{j}}R_{rj}$ and $\lambda_{ij}^{INT_{j}}INT_{rtj}$. Additionally defining $T_{t1j}=T_{tj}$ gives:

(5) $$\tau_{rt1j}=\mu_{1j}+T_{tj}+R_{rj}+INT_{rtj}$$

for the reference (standard) indicators and

(6) $$\tau_{rtij}=\mu_{ij}+\lambda_{ij}^{T_{j}}T_{tj}+\lambda_{ij}^{R_{j}}R_{rj}+\lambda_{ij}^{INT_{j}}INT_{rtj}+OM_{tij}$$

for non-reference (non-standard) indicators.

Our final model equations for observed variables are:

(7) $$Y_{rt1j}=\mu_{1j}+T_{tj}+R_{rj}+INT_{rtj}+\varepsilon_{rt1j}$$

and

(8) $$Y_{rtij}=\mu_{ij}+\lambda_{ij}^{T_{j}}T_{tj}+\lambda_{ij}^{R_{j}}R_{rj}+\lambda_{ij}^{INT_{j}}INT_{rtj}+OM_{tij}+\varepsilon_{rtij}.$$

Note that all latent variables on the target-level (i.e., all latent trait-variables and all object-specific method-effect variables) reflect parts of true expected ratings that are shared (“common perspective”) by all raters (Koch et al. 2016; see also Eid et al. 2008). Note further that all latent variables within an equation are orthogonal. $T_{tj}$, $R_{rj}$ and $INT_{rtj}$ are orthogonal due to the cross-classified data structure in which the target- and rater-levels are independent (given that the set of targets and the set of raters do not contain the same persons) and the interaction-level encompasses residual variables after the variables on the higher levels are controlled for (see also Koch et al. 2016). The $OM_{tij}$ are orthogonal to $T_{tj}$ as they depict DT-object (e.g., AUT-object) specific residuals to the conditional expectation given the reference DT-object. The following covariances are included: $\sigma_{T_{j}T_{j^{\prime }}}$ depicts the covariance of two traits across two different constructs (*j* ≠ *j′*) and reflects discriminant validity from an MTMM-perspective. $\sigma_{OM_{ij}OM_{i^{\prime }j^{\prime }}}$ depicts the covariance of two different DT-object method-effects that may relate to different objects (*i* ≠ *i′*) but the same construct (*j* = *j′*), the same object (*i* = *i′*) but different constructs (*j* ≠ *j′*), or different objects (*i* ≠ *i′*) and different constructs (*j* ≠ *j′*). $\sigma_{R_{j}R_{j^{\prime }}}$ depicts the covariance of the two rater-effects across two different constructs and $\sigma_{INT_{j}INT_{j^{\prime }}}$ depicts the covariance of two interaction-effects across two different constructs. Note that $\sigma_{R_{j}R_{j^{\prime }}}$ and $\sigma_{INT_{j}INT_{j^{\prime }}}$ can only be estimated if the same raters (at least in part) are employed across rating procedures (constructs) which is not always the case in DT-studies (e.g., Forthmann et al. 2017). 

The DTCC can be simplified to the DTTL by conglomerating the rater-effect and the interaction-effect for each construct into a unique (referencing rater-disagreement or uniqueness; Eid et al. 2008; Koch et al. 2016) method-effect ($UM_{rtj}$):

(9) $$\lambda_{ij}^{R_{j}}R_{rj}+\lambda_{ij}^{INT_{j}}INT_{rtj}=\lambda_{ij}^{UM_{j}}UM_{rtj},$$

with $\lambda_{1j}^{R_{j}}=\lambda_{1j}^{INT_{j}}=\lambda_{1j}^{UM_{j}}=1$. This would be necessary if every target had her or his unique set of raters, i.e., the data structure would not be cross-classified but adhere to a standard two-level sampling process (Eid et al. 2008). Even in the case of cross-classified data, the simplified model may be used if the separation of raters and rater–target interactions is not of particular interest as long as parameter estimates do not undergo substantial bias and affect the variance decomposition which we will explore in the empirical application. In Figure 2, names and labels in parentheses show the appropriate names and labels for latent variables and parameters on the lower level (level-1) of the DTTL (in which the target-level is the only higher level or level-2).

## 3. Variance Decomposition

Due to the orthogonalized structure of latent variables within an observed variable, the variance decomposition of the DTCC is straightforward. The complete variances for any standard indicator (*i* = 1) and any non-standard indicator (*i* ≠ 1) of construct *j* are, respectively, given by

(10) $$\sigma_{Y_{rt1j}}^{2}=\sigma_{T_{tj}}^{2}+\sigma_{R_{rj}}^{2}+\sigma_{INT_{rtj}}^{2}+\sigma_{\varepsilon_{rt1j}}^{2}$$

and

(11) $$\sigma_{Y_{rtij}}^{2}={\left(\lambda_{ij}^{T_{j}}\right)}^{2}\sigma_{T_{tj}}^{2}+{\left(\lambda_{ij}^{R_{j}}\right)}^{2}\sigma_{R_{rj}}^{2}+{\left(\lambda_{ij}^{INT_{j}}\right)}^{2}\sigma_{INT_{rtj}}^{2}+\sigma_{OM_{tij}}^{2}+\sigma_{\varepsilon_{rtij}}^{2},$$

with the variance of the respective true score encapsulating all components except for measurement error (residual):

(12) $$\sigma_{\tau_{rt1j}}^{2}=\sigma_{T_{tj}}^{2}+\sigma_{R_{rj}}^{2}+\sigma_{INT_{rtj}}^{2}=\sigma_{Y_{rt1j}}^{2}-\sigma_{\varepsilon_{rt1j}}^{2}$$

and

(13) $$\sigma_{\tau_{rtij}}^{2}={\left(\lambda_{ij}^{T_{j}}\right)}^{2}\sigma_{T_{tj}}^{2}+{\left(\lambda_{ij}^{R_{j}}\right)}^{2}\sigma_{R_{rj}}^{2}+{\left(\lambda_{ij}^{INT_{j}}\right)}^{2}\sigma_{INT_{rtj}}^{2}+\sigma_{OM_{tij}}^{2}=\sigma_{Y_{rtij}}^{2}-\sigma_{\varepsilon_{rtij}}^{2}.$$

For the true scores of standard indicators, we can define the following meaningful relative variance parameters. The model-implied ICC ($MIICC_{1j}$) depicts the proportion of true score variance that is attributable to variability in the targets:

(14) $$MIICC_{1j}=\frac{\sigma_{T_{tj}}^{2}}{\sigma_{T_{tj}}^{2}+\sigma_{R_{rj}}^{2}+\sigma_{INT_{rtj}}^{2}}.$$

It can be interpreted as convergent validity from an MTMM perspective. The rater (method) specificity coefficient ($RMS_{1j}$) depicts the proportion of true score variance that is attributable to variability in the raters:

(15) $$RMS_{1j}=\frac{\sigma_{R_{rj}}^{2}}{\sigma_{T_{tj}}^{2}+\sigma_{R_{rj}}^{2}+\sigma_{INT_{rtj}}^{2}}.$$

The interaction (method) specificity coefficient ($IMS_{1j}$) depicts the proportion of true score variance that is attributable to variability in rater–target interactions:

(16) $$IMS_{1j}=\frac{\sigma_{INT_{rtj}}^{2}}{\sigma_{T_{tj}}^{2}+\sigma_{R_{rj}}^{2}+\sigma_{INT_{rtj}}^{2}}.$$

One may also calculate a unique method specificity coefficient ($UMS_{1j}$) that depicts the proportion of true score variance that is not attributable to targets but to any kind of rater-related method-effect:

(17) $$UMS_{1j}=\frac{\sigma_{R_{rj}}^{2}+\sigma_{INT_{rtj}}^{2}}{\sigma_{T_{tj}}^{2}+\sigma_{R_{rj}}^{2}+\sigma_{INT_{rtj}}^{2}}=1-MIICC_{1j}.$$

For the non-standard indicators, the following meaningful relative variance parameters can be defined. The level-2 (target-level) consistency coefficient ($L2Con_{ij}$) shows the proportion of target-variability in a non-standard indicator (non-standard object) that can be explained by the target-variability of the standard indicator (standard object):

(18) $$L2Con_{ij}=\frac{{\left(\lambda_{ij}^{T_{j}}\right)}^{2}\sigma_{T_{tj}}^{2}}{{\left(\lambda_{ij}^{T_{j}}\right)}^{2}\sigma_{T_{tj}}^{2}+\sigma_{OM_{tij}}^{2}}.$$

In contrast to this, the level-2 object-method specificity coefficient ($L2OMS_{ij}$) depicts the remaining unexplained proportion of target-level variance that is attributable to object method-effects:

(19) $$L2OMS_{ij}=\frac{\sigma_{OM_{tij}}^{2}}{{\left(\lambda_{ij}^{T_{j}}\right)}^{2}\sigma_{T_{tj}}^{2}+\sigma_{OM_{tij}}^{2}}=1-L2Con_{ij}.$$

Both standard object consistency and non-standard object specificity can also be computed for the overall true score variability, giving the level-1 consistency coefficient ($L1Con_{ij}$) and the level-1 object-method specificity coefficient ($L1OMS_{ij}$), respectively:

(20) $$L1Con_{ij}=\frac{{\left(\lambda_{ij}^{T_{j}}\right)}^{2}\sigma_{T_{tj}}^{2}}{\sigma_{\tau_{rtij}}^{2}}$$

and

(21) $$L1OMS_{ij}=\frac{\sigma_{OM_{tij}}^{2}}{\sigma_{\tau_{rtij}}^{2}}$$

The rater specificity coefficient, the interaction specificity coefficient and the unique method specificity coefficient may also be defined for the non-standard indicators:

(22) $$RMS_{ij}=\frac{{\left(\lambda_{ij}^{R_{j}}\right)}^{2}\sigma_{R_{rj}}^{2}}{\sigma_{\tau_{rtij}}^{2}},$$

(23) $$IMS_{ij}=\frac{{\left(\lambda_{ij}^{INT_{j}}\right)}^{2}\sigma_{INT_{rtj}}^{2}}{\sigma_{\tau_{rtij}}^{2}},$$

and

(24) $$UMS_{ij}=\frac{{\left(\lambda_{ij}^{R_{j}}\right)}^{2}\sigma_{R_{rj}}^{2}+{\left(\lambda_{ij}^{INT_{j}}\right)}^{2}\sigma_{INT_{rtj}}^{2}}{\sigma_{\tau_{rtij}}^{2}}$$

Lastly, the model-implied ICC for non-standard indicators must respect both the target-specific trait-variance and the target-specific object method-effect variance:

(25) $$MIICC_{ij}=\frac{{\left(\lambda_{ij}^{T_{j}}\right)}^{2}\sigma_{T_{tj}}^{2}+\sigma_{OM_{tij}}^{2}}{\sigma_{\tau_{rtij}}^{2}}=1-UMS_{ij}.$$

For all indicators, regardless of whether they are standard indicators or not, we can, of course, compute reliability ($REL_{ij}$) as the proportion of true score variance in the complete variance:

(26) $$REL_{ij}=\frac{\sigma_{\tau_{rtij}}^{2}}{\sigma_{Y_{rtij}}^{2}}=1-\frac{\sigma_{\varepsilon_{rtij}}^{2}}{\sigma_{Y_{rtij}}^{2}}.$$

Please note that the consistency- and method specificity-coefficients for non-standard indicators reflect direct adaptations of the respective coefficients for the original C4 (see Equations (21)–(25) in (Koch et al. 2016). Within the DTCC, $RMS_{ij}$, $IMS_{ij}$, and $UMS_{ij}$ are also definable for the standard indicators/objects (*i* = 1) because they employ interchangeable raters as well.

For the DTTL, the variance decomposition is given in detail in Appendix A. Essentially, for this simplified model, Equations (10)–(26) hold with the following restrictions: $\lambda_{ij}^{INT_{j}}=\lambda_{ij}^{UM_{j}}$, $\sigma_{INT_{rtj}}^{2}=\sigma_{UM_{rtj}}^{2}$, $IMS_{ij}=UMS_{ij}$, and $\lambda_{ij}^{R_{j}}=\sigma_{R_{rj}}^{2}=RMS_{ij}=0$.

## 4. Empirical Application

We illustrate the DTCC and the DTTL in comparison on an openly accessible dataset (available at: https://osf.io/a9qnc, accessed on 20 July 2024) containing various forms of AUT ratings. We first describe the data and its structure, then lay out the analytic strategy and finally present the results.

### 4.1. The Data

The data stem from a DT-study executed in Germany and has been analyzed several times before (Forthmann et al. 2017, 2020; Forthmann and Doebler 2022). 202 target-participants (144 reported to be female; mean of age = 25.51, standard deviation of age = 6.813, range of age: 17 to 75) received “be-creative” instructions for the three AUTs of rope (*i* = 1), garbage bag (*i* = 2), and paperclip (*i* = 3). The time limit for the idea production phase for each AUT was 2.5 min. There were seven raters for the derivation of scores. Raters 1 through 5 gave snapshot cleverness (*j* = 1) ratings for each set of produced ideas. Rater-instructions stated that highly clever ideas should be imaginative, apt, ingenious, and funny, whereas unclever ideas should be too vague, too general, negligibly relevant, and without sophistication. Raters 2, 6, and 7 judged every individual idea with regard to creative quality (*j* = 2). For each creative quality rating, the raters were instructed to weigh the aspects of uncommonness, remoteness, and cleverness against each other so that high-quality ideas would strongly represent all three aspects. Both cleverness and creative quality ratings were given on a 5-point Likert scale (range: 1 to 5). For the creative quality ratings, we computed the 0.75 quantile of the set of produced ideas for each rater–target dyad on each AUT and used these values as scores for the analysis. Usage of the 0.75 quantile (instead of the median or mean) is common since it is more robust against low-quality outlier ideas of otherwise very creative targets (e.g., Forthmann et al. 2017, 2019). Note that only one rater (rater 2) gave both cleverness and creative quality ratings. This is not enough to estimate the rater-effect covariance ($\sigma_{R_{1}R_{2}}$) in the DTCC and unsatisfactory for estimating the interaction-effect covariance ($\sigma_{INT_{1}INT_{2}}$) and the rater method-effect covariance ($\sigma_{RM_{1}RM_{2}}$) in the DTCC and DTTL, respectively. Thus, we excluded rater 2 from the cleverness ratings and orthogonalized all rater-related effects (i.e., we used the ratings of rater 1, 3, 4, and 5 for cleverness and the ratings of rater 2, 6, and 7 for creative quality).

These measures constitute the observed variables for our main analysis. The average measure absolute agreement ICC is 0.906 (95% confidence interval: [0.882, 0.925]) for cleverness and 0.479 (95% confidence interval: [0.403, 0.547]) for creative quality. Table 1 shows an excerpt of the cross-classified data. The Supplementary Materials contains an R-script (R Core Team 2013) that shows how to download the data from https://osf.io/a9qnc and then restructure it in the required way. For further information on the complete data of the study, we refer to Forthmann et al. (2017).

### 4.2. Analytic Strategy

In both models, the AUT-object of rope (*i* = 1) was chosen as the reference method for both cleverness and creative quality. We believe that, out of the given three objects, the rope (together with similar objects like the string) probably has the longest history and is the most used as an AUT-object (e.g., the string was used as an illustrative AUT example in Wallach and Kogan 1965). Therefore, it can be seen as a “gold-standard” (Eid et al. 2003) and the DTCC (and DTTL) allows exploration of the object-specificity of newer AUTs in comparison to this long-lasting standard.

To the best of our knowledge, a maximum likelihood estimator for cross-classified CFAs containing freely estimated factor-loadings (such as the C4 and the DTCC) has not yet been derived (see Jeon and Rijmen 2014 and Koch et al. 2016 for discussions). Thus, we estimated the DTCC and the DTTL using Bayesian Markov Chain Monte Carlo simulation with three Gibbs sampling chains. Appendix B shows the prior specifications in both models. Further, we also estimated the DTTL using full information maximum likelihood with robust Huber–White standard errors (e.g., Long and Ervin 2000) in order to compare the Bayesian estimator with a frequentist approach. In the remainder, the DTTL estimated with Bayesian statistics will be referred to as the DTTL-B and the DTTL estimated with the maximum-likelihood method will be referred to as the DTTL-ML.

For the Bayesian estimation, we set the standard Gelman–Rubin convergence criterion of a maximum potential scale reduction (PSR) factor of 1.1 (Asparouhov and Muthén 2021; Gelman and Rubin 1992; Muthén and Muthén 1998–2017). The DTCC converged after 64,998 iterations, the DTTL-B converged after 21,898 iterations. The first half of the iterations was treated as burn-in. For an additional convergence check, we created trace plots which are presented in the Supplementary Materials. These were unsuspicious with the exception that the trace plots for the creative quality rater-effect variance in the DTCC indicated problems. Therefore, we re-estimated the DTCC with a fixed number of 1,000,000 iterations, with the first half treated as burn-in. The estimation time was 87 min on a 3.4 GHz processor personal computer, the highest PSR factor was 1.021 and any observed differences in comparison to the Gelman–Rubin procedure (see Supplementary Materials) did not affect the main conclusions. The results of the fixed iterations procedure will be reported in the following.

For the DTCC and the DTTL-B, we investigated model fit with the Bayesian Posterior Predictive Checking procedure (BPPC; e.g., Asparouhov and Muthén 2021; Gelman et al. 1996) using the $\chi^{2}$-statistics. A model is maintained when the 95% confidence interval (COI) for the difference between the observed and the replicated $\chi^{2}$-values ($\Delta \chi^{2}$) contains zero and the posterior predictive *p*-value is above .05. For the DTTL-ML, we investigated the $\chi^{2}$-test of exact fit, the RMSEA, and the SRMR for level-1 and level-2 with regard to standard cut-off values. A model is rejected if the *p*-value of the $\chi^{2}$-test statistic is below .05. Model fit is considered acceptable if RMSEA, SRMR_level-1_, and SRMR_level-2_ are equal to or below .08 and good if they are equal to or below .05 (Browne and Cudeck 1992; Chen et al. 2008; Hu and Bentler 1999; West et al. 2012). We will compare the parameters and relative variances across the three models. For all analyses, we used version 8.7 of Mplus (Muthén and Muthén 1998–2017). The Supplementary Materials contains Mplus-scripts (Input files) and results (Output files).

### 4.3. Results and Discussion

The DTCC did not have to be rejected (*M*($\Delta \chi^{2}$) = −4.438, 95%-COI = [−56.534, 64.942], *p* = .438) and neither did the DTTL-B (*M*($\Delta \chi^{2}$) = −2.127, 95%-COI = [−28.479, 33.249], *p* = .442). The DTTL-ML was rejected based on the test of exact fit and yielded a high RMSEA, but the SRMR statistics suggested good model fit ($\chi^{2}$(13) = 207.954, *p* < .001, RMSEA = 0.103, SRMR_level-1_ = 0.013, SRMR_level-2_ = 0.040).

Table 2 shows the complete results for the parameters and relative variances of all three models. All parameter estimates were positive and no 95% CRI or COI (in the case of the DTTL-ML) contained zero—with the exception of two non-significant object method-effect covariances in the DTTL-ML. Overall, the models yielded quite similar results (with some exceptions). On the target-level, more variance was observed in the latent trait for cleverness (around 0.570) than for creative quality (around 0.200). Within the DTCC, rater- and interaction-effects were quite sparse and balanced for cleverness ($\sigma_{R_{r1}}^{2}$ = 0.055, $\sigma_{INT_{rt1}}^{2}$ = 0.047). For creative quality, overall more true score variability was attributable to unique method-effects and differences in raters were much more pronounced than differences in rater–target interactions ($\sigma_{R_{r2}}^{2}$ = 0.328, $\sigma_{INT_{rt2}}^{2}$ = 0.004) which suggests that, while raters differed in their judgements, each rater was consistent across the targets. Within the two DTTL models, unique method-effect variance was much higher for creative quality than for cleverness as well (DTTL-B: $\sigma_{UM_{r1}}^{2}$ = 0.084, $\sigma_{UM_{r2}}^{2}$ = 0.253; DTTL-ML: $\sigma_{UM_{r1}}^{2}$ = 0.109, $\sigma_{UM_{r2}}^{2}$ = 0.240). With regard to trait correlation, some important differences between the models need to be mentioned. Trait correlation was estimated to be high in all models but substantially higher for the DTTL, especially with the maximum likelihood-estimator (DTCC: $r_{T_{1}T_{2}}$ = 0.876, DTTL-B: $r_{T_{1}T_{2}}$ = 0.941, DTTL-ML: $r_{T_{1}T_{2}}$ = 0.994). Analyzing the data with a standard CFA as depicted in Figure 1, Forthmann et al. (2017) found a (first-order) latent correlation between cleverness and creative quality of 0.831 which comes closest to our result for the DTCC (it must be mentioned that their CFA model included several more latent and observed variables, that they had one rater and two targets less, and that their computation for creative quality scores was different which will be discussed further below). Object method-effect correlations were also very high in all models of the current application when the two object method-effects related to the same object but different constructs (around 0.900).

For the relative variances, again, results were very similar across models with relative variance parameters usually not differing by more (and often less) than 5 percentage points between models. The highest difference was observed for the reliability of the first AUT (rope) scored for creative quality. This difference amounted to 12.5 percentage points and was found between the DTCC (*REL*_12_ = 0.780) and the DTTL-ML (*REL*_12_ = 0.655). Across all models, constructs, and AUTs, the level-2 consistency was rather low (ranging from 0.188 to 0.259), displaying a large amount of level-2 object-method specificity (ranging from 0.741 to 0.812, accordingly). Level-1 object-method specificity was also larger than level-1 standard object consistency. Note that, from an MTMM perspective, high method specificity is reflective of low convergent validity and highly correlated traits (see above) are reflective of low discriminant validity. Within the DTCC, rater-specificity and interaction-specificity were quite low for cleverness (range: *RMS*_31_ = 0.022 to *RMS*_11_ = 0.080). For creative quality, interaction-specificity was very low (range: *IMS*_22_ = 0.004 to *IMS*_32_ = 0.009) but rater-specificity was much higher (range: *RMS*_32_ = 0.321 to *RMS*_12_ = 0.590) which, again, nicely displays substantial differences between the raters, on one hand, but strong rater-consistency across targets, on the other hand. Thus, unique method specificity was rather low for cleverness but substantial for creative quality which was also found in similar quantities for the DTTL regardless of the estimator. Accordingly, across all three models, the model implied ICCs were very high for cleverness but much lower for creative quality. Within the DTCC, we found for cleverness: *MIICC*_11_ = 0.846, *MIICC*_21_ = 0.885, and *MIICC*_31_ = 0.911; and for creative quality: *MIICC*_12_ = 0.401, *MIICC*_22_ = 0.405, and *MIICC*_32_ = 0.663. We can, again, compare these results to Forthmann et al. (2017) who computed average measure absolute agreement ICCs before the modeling. For cleverness, they found an ICC of 0.849. We yielded slightly higher results, which is partially explainable by slightly different data (see above), but also by the fact that the ICC of the current contribution relies on model assumptions, as well as by the fact that it only considers true score variance and excludes measurement error. For creative quality, Forthmann et al. (2017) found an ICC of 0.711. This is substantially higher than our results. We believe that this is largely explainable by the fact that Forthmann et al. (2017) computed the ICC across all individual idea ratings and then employed a different procedure for retrieving a target score out of the individual idea ratings. They first averaged the individual idea ratings for each rater–target dyad on each AUT. Then, they used the 0.75 quantile of the distribution over the three raters as the actual score for the CFA. We first took the 0.75 quantile of the distribution over individual idea ratings on each AUT for each rater–target dyad and then aggregated across the raters within the CFA models. Therefore, our model-implied ICCs refer to 0.75 quantile scores (instead of mean scores) that actually entered the model. We argue that our procedure better reflects the rationale for the 0.75 quantile, as put forward by Forthmann et al. (2017):

For example, there could be two participants who have the same number of good quality ideas, but one of the two has several more low-quality ideas. On average, these two performances may differ a great deal, but if the upper tails of their distributions are considered, the performances of both persons are much more alike.

(p. 261)

Thus, when low-quality ideas within a set of ideas should receive less weight in computing the score, the unweighted average across ideas should be avoided and the 0.75 quantile should be used for the distribution over the ideas (not raters).

In any case, low rater consent (represented by high unique method specificities and low model-implied ICCs) is unfortunate as it minimizes the (trait) variability across targets. A minimized variance also minimizes potential covariance, which is a problem if the latent variables on the target-level were to be used as predictors, for example. Nevertheless, the DTCC (and the DTTL) still appropriately aggregates the target-specific variance from the total variance.

Lastly, the indicators (AUT-tasks) were quite reliable across models and constructs as the latent variables were able to explain more than 50% of the total variance in any case. Reliabilities ranged from DTTL-ML: *REL*_32_ = 0.559 to DTCC: *REL*_12_ = 0.780.

## 5. General Discussion

In this contribution, we showed how the Correlated Traits Correlated Methods Minus One Multitrait–Multimethod model for cross-classified data (Koch et al. 2016) can be modified and applied in typical creativity research scenarios where an independent set of raters judge the creative ideation of study targets. The model can be used to analyze the variance decomposition of divergent thinking tasks (such as alternate use tasks, but the model is not limited to those) in which raters assess various aspects of creativity in ideas produced by target subjects. The model is strongly grounded in classical test theory and stochastic measurement theory which gives its latent variables clear meaning. It does not only separate true scores from measurement error but also decomposes the true score into target-specific traits and DT-object-specific, rater-specific, and rater–target interaction-specific method-effects. Thus, it allows for computing true score variance proportions attributable to the various levels of the cross-classified data structure and objects of specific divergent thinking problems.

### 5.1. Substantive Deliberations

The high object-method specificity and the high trait-correlation we found in the empirical application is something important to consider. Compare the measurement of DT to the measurement of personality. Within a Big 5 (Costa and McCrae 1989) personality trait like conscientiousness, for example, different indicators are supposed to measure structurally different facets or aspects (DeYoung et al. 2007) of the overarching personality construct. For instance, the indicator “I keep things tidy” measures the conscientiousness-aspect of orderliness while the indicator “I get things done quickly” measures the conscientiousness aspect of industriousness. Within DT, one would expect cleverness and creative quality (or, more fine-grained, remoteness or uncommonness, for example) to reflect structurally different aspects of DT, but one would consider different AUT-objects (such as rope, garbage bag, or paperclip) as interchangeable methods for assessing an aspect. Our empirical results, however, suggest that this is not the case. The cross-construct correlation among the trait variables was considerably higher than the reference-object consistency coefficients within a single construct which reflects a simultaneous lack of both convergent and discriminant validity of the creativity aspects. Thus, one might ask whether different AUT-objects (maybe also DT-objects in general) may actually differ in their representation of a specific creativity aspect or facet. For example, do ideas for ropes (such as using a single rope fiber as dental floss) better correspond to remoteness, whereas ideas for paperclips (such as building a humanoid sculpture with them) better correspond to cleverness? In this regard, we shall, however, note that the high cross-construct correlations were expected since creative quality raters were instructed to also consider cleverness (cleverness was a confound in creative quality). The DTCC is probably put to its best use when different aspects of creativity (cleverness, remoteness, uncommonness, usefulness, etc.) are to be scrutinized separately. We note that even when high object-method-specificity is present, the trait and object-specific method-effects of a single construct can be used in an efficient manner for criterion prediction (e.g., for predicting creative achievement).

### 5.2. Modifications, Extensions, Useful Applications, and Limitations of the Model

The subjectivity component of creativity will certainly remain a problem for assessing it with scientific methods that aim at objectivity. After all, the phrase “creative differences” that is often used to explain the parting of formerly aligned artists exists for a reason. The DTCC can be expanded to contain explanatory variables for rater-effects and rater–target interactions-effects. For example, concerning variability among raters, it has been found that art experts often perceive artistic works differently than laymen (e.g., Pihko et al. 2011; Vogt and Magnussen 2007). Concerning variability among rater–target interactions, one could investigate whether a fit between target and rater with regard to a self-reported preference of a specific creativity aspect (e.g., remoteness vs. funniness) explains interaction-effects. In the empirical illustration of the current contribution, however, we found only little variability in interactions, suggesting that raters were consistent with their rating standards across targets. In this regard, it must be stated that rater instructions are usually given to raters in DT-studies not least to minimize such subjectivity; however, how the remaining subjectivity can be explained is—to the best of our knowledge—still an open question.

The DTCC (and the DTTL) can be expanded and modified to include all kinds of DT-scores and break down the variance in various ways. For example, typing speed and/or fluency could be included as target-level specific (latent) variables and could be partialized out of the measurement of creative quality (see also Forthmann et al. 2017). In this vein, the CTC(M − 1) logic can be further applied. For example, a fluency trait variable could be conceptualized as a trait assessed with a reference scoring method and a cleverness latent variable could be conceptualized as a latent non-reference scoring method residual variable (see Forthmann et al. 2019 for a similar approach using completely structurally different indicators in a standard CFA). Note that this particular example would demand the use of the original C4 as displayed in Koch et al. (2016) since fluency only varies across the targets but cleverness varies on the target-, rater-, and interaction-level. This approach would, however, not be recommended when the different scoring methods are highly correlated (which was the case for cleverness and creative quality in the current contribution) as the residual scoring method variance would be expected to be very low. This potentially causes anomalous results (or at least results that are difficult to interpret) such as irregular loading patterns and high standard errors (see Jendryczko and Nussbeck 2024a).

We would like to stress that raters (and targets) within the DTCC (and the original C4) and the DTTL are regarded as interchangeable, meaning they are conceptualized as outcomes of a random variable. We already discussed that predictor variables can be used to explore any structural differences between raters and between rater–target interactions. Yet, one might also want to test the interchangeability assumption without the presence of any predictors. To the best of our knowledge, this is not possible within the setting of cross-classified CFAs. It is, however, possible to test the interchangeability of rater–target interactions within a Two-Level CFA (and thus within the DTTL) using the wide data format approach (Curran 2003; Mehta and Neale 2005; Nussbeck et al. 2009; see also Jendryczko and Nussbeck 2022, 2024b). In this approach, there exists no rater-variable as a column in the data frame, but each rater is represented by a separate column. Level-2 of the model needs to be extended in a certain way and every latent variable on Level-1 needs to specified once for every rater–target interaction. By implementing certain equality constraints (see, for example, Nussbeck et al. 2009) the model is made parametrically identical to the DTTL as presented in the current contribution. Using maximum likelihood estimation, the unconstrained and the constrained model can be compared with a likelihood-ratio test to test the Null hypothesis that both models explain the observed means and covariances equally well. If the Null hypothesis can be maintained, interchangeability can be assumed (Jendryczko and Nussbeck 2022).

The question remains how well the presented models and their estimation procedures recover the true parameters. Importantly, the estimation of the rater-effect variance for creative quality was somewhat unstable with regard to convergence (see Supplementary Materials). This might be due to the very small sample of raters. There were only three raters for creative quality; however, note that the estimation of the rater-effect variance for cleverness worked much better even though there was only one rater more for this construct. Thus, we recommend employing at least four, if not five raters for every creativity aspect in future applications. With the empirical illustration, we showed that Bayesian estimation of the DTCC, Bayesian estimation of the DTTL and maximum-likelihood estimation of the DTTL deliver similar results and lead to the same general conclusions (at least in this specific application). However, there were some notable differences, such as a higher trait-correlation within the DTTL (especially using maximum likelihood) and some (albeit only a few) substantial differences in relative variances. Which model recovers the true parameters best? This can only be answered with simulation studies. We hypothesize that, given that the DTCC is the data generating model, the DTCC recovers the relative variances best, but the DTTL can still sufficiently recover relative variances in most cases. We shall also note that we treated the ratings as continuous, but it should be possible to derive variants of the models for ordered categorical data (see also Nussbeck et al. 2006). This has particular relevance for typical rating procedures in DT-studies, as one could argue that the usual discrete rating scales (e.g., 1, 2, 3, 4, and 5 as possible outcomes in the application of this contribution) are better treated as ordinal rather than continuous.

## 6. Conclusions

The Divergent Thinking Cross-Classified model as a modification of the Correlated Traits Correlated Methods Minus One model for cross-classified data is a useful tool for modeling the rated responses to divergent thinking tasks as the model is able to take all variance components into account. Future studies need to derive it for ordered categorical data and investigate its statistical properties with simulation studies. Its foundation in structural equation modeling enables flexible extensions and modifications for pursuing new research objectives.

## Figures and Tables

**Table 1:** Cross-classified structure of the dataset as used in the analysis.

Target	Rater	*Y*_11_	*Y*_21_	*Y*_31_	*Y*_12_	*Y*_22_	*Y*_32_
1	1	3	3	2	NA	NA	NA
1	2	NA	NA	NA	3.00	2.25	3.00
1	3	3	2	2	NA	NA	NA
1	4	4	3	3	NA	NA	NA
1	5	4	3	3	NA	NA	NA
1	6	NA	NA	NA	4.00	5.00	3.00
1	7	NA	NA	NA	2.00	3.00	3.00
2	1	2	4	3	NA	NA	NA
2	2	NA	NA	NA	3.00	3.50	1.75
2	3	2	4	2	NA	NA	NA
2	4	3	4	2	NA	NA	NA
2	5	3	4	2	NA	NA	NA
2	6	NA	NA	NA	4.00	4.50	2.75
2	7	NA	NA	NA	2.00	3.50	2.00

Notes. *Y_ij_* = rating for AUT-object (indicator) *i* (1 = rope, 2 = garbage bag, 3 = paperclip) with regard to construct *j* (1 = cleverness, 2 = creative quality), NA = Not Available (missing value). There were 202 targets in total but only two are shown.

**Table 2:** Parameter estimates and relative variances for all models.

			DTCC						DTTL-B						DTTL-ML			
Parameter	*Y*_11_	*Y*_21_	*Y*_31_	*Y*_12_	*Y*_22_	*Y*_32_	*Y*_11_	*Y*_21_	*Y*_31_	*Y*_12_	*Y*_22_	*Y*_32_	*Y*_11_	*Y*_21_	*Y*_31_	*Y*_12_	*Y*_22_	*Y*_32_
$\mu_{ij}$	2.993	3.066	3.026	2.907	3.132	2.927	2.996	3.055	3.024	2.908	3.124	2.922	2.993	3.069	3.030	2.904	3.129	2.926
$\lambda_{ij}^{T_{j}}$	1	0.489	0.498	1	0.540	0.459	1	0.510	0.514	1	0.531	0.461	1	0.521	0.508	1	0.598	0.497
$\lambda_{ij}^{R_{j}}$	1	0.715	0.502	1	1.108	0.583	-	-	-	-	-	-	-	-	-	-	-	-
$\lambda_{ij}^{INT_{j}}$ $\;\mathrm{or}\;\lambda_{ij}^{UM_{j}}$	1	0.997	0.908	1	0.932	1.004	1	0.917	0.762	1	1.114	0.596	1	0.775	0.638	1	1.163	0.613
$\sigma_{\varepsilon_{rtij}}^{2}$	0.324	0.261	0.273	0.158	0.209	0.188	0.346	0.257	0.274	0.183	0.207	0.208	0.321	0.262	0.275	0.208	0.195	0.209
$\sigma_{T_{tj}}^{2}$	0.588			0.223			0.564			0.195			0.579			0.156		
$\sigma_{OM_{tij}}^{2}$		0.468	0.429		0.210	0.182		0.469	0.424		0.207	0.180		0.456	0.426		0.170	0.137
$\sigma_{R_{rj}}^{2}$	0.055			0.328			-			-			-			-		
$\sigma_{INT_{rtj}}^{2}$ $\;\mathrm{or}\;\sigma_{UM_{rtj}}^{2}$	0.047			0.004			0.084			0.253			0.109			0.240		
$\sigma_{T_{1}T_{2}}$			0.316 (.876)						0.310 (.941)						0.298 (.994)			
$\sigma_{OM_{21}OM_{31}}$			0.187 (.420)						0.182 (.411)						0.181 (.410)			
$\sigma_{OM_{21}OM_{22}}$			0.285 (.912)						0.282 (.908)						0.162 (.942)			
$\sigma_{OM_{21}OM_{32}}$			0.075 (.260)						0.079 (.274)						0.058 (.232)			
$\sigma_{OM_{31}OM_{22}}$			0.073 (.245)						0.066 (.224)						0.054 (.200) *			
$\sigma_{OM_{31}OM_{32}}$			0.251 (.901)						0.251 (.913)						0.231 (.958)			
$\sigma_{OM_{22}OM_{32}}$			0.051 (.263)						0.049 (.259)						0.007 (.045) *			
*L*2*Con_ij_*		.231	.254		.237	.205		.238	.259		.210	.188		.256	.259		.247	.219
*L*2*OMS_ij_*		.769	.746		.763	.795		.762	.741		.790	.812		.744	.741		.753	.781
*L*1*Con_ij_*		.201	.228		.091	.126		.213	.238		.095	.133		.232	.241		.101	.145
*L*1*OMS_ij_*		.672	.674		.306	.520		.681	.680		.357	.575		.672	.688		.310	.515
*MIICC_ij_*	.846	.885	.911	.401	.405	.663	.870	.897	.921	.435	.457	.714	.842	.904	.928	.394	.411	.660
*RMS_ij_*	.080	.041	.022	.590	.589	.321	-	-	-	-	-	-	-	-	-	-	-	-
*IMS_ij_*	.065	.065	.060	.006	.004	.009	-	-	-	-	-	-	-	-	-	-	-	-
*UMS_ij_*	.154	.115	.089	.599	.595	.337	.130	.103	.079	.565	.543	.286	.158	.096	.072	.606	.589	.340
*REL_ij_*	.688	.730	.701	.780	.767	.655	.654	.730	.697	.710	.738	.602	.680	.721	.693	.655	.738	.559

Notes. *N* = 202. DTCC = Divergent Thinking Cross-Classified Model (Bayesian estimator with a fixed number of 1,000,000 iterations), DTTL-B = Divergent Thinking Two-Level Model estimated using Bayesian statistics (Gelman–Rubin criterion), DTTL-ML = Divergent Thinking Two-Level Model estimated using full information maximum likelihood (robust standard errors). *Y_ij_* = AUT-score variable of object *i* (1 = rope, 2 = garbage bag, 3 = paperclip) scored for construct *j* (1 = cleverness, 2 = creative quality). *μ* indicates an intercept, *λ* indicates a factor-loading, *σ*^2^ indicates a variance, and *σ* indicates a covariance. *ε_rtij_* = residual of an AUT-score variable, *T_tj_* = latent trait variable for construct *j*, *OM_tij_* = (DT-) object-specific method-effect variable for non-reference object *i* for construct *j*, *R_rj_* = rater-effect variable for construct *j*, *INT_rtj_* = interaction-effect variable for construct *j*, *UM_rtj_* = unique method-effect variable for construct *j*, *L*2*Con_ij_* = level-2 consistency for non-reference object *i* of construct *j*, *L*2*OMS_ij_* = level-2 object-method specificity for non-reference object *i* of construct *j*, *L*1*Con_ij_* = level-1 consistency for non-reference object *i* of construct *j*, *L*1*OMS_ij_* = level-1 object-method specificity for non-reference object *i* of construct *j*, *MIICC_ij_* = model-implied intra-class correlation of indicator *i* for construct *j*, *RMS_ij_* = rater specificity of indicator *i* for construct *j*, *IMS_ij_* = interaction specificity of indicator *i* for construct *j*, *UMS_ij_* = unique method specificity of indicator *i* for construct *j*, *REL_ij_* = reliability of indicator *i* for construct *j*. Values of 1 were fixed. Numbers in parentheses depict correlations. Two-sided 95%-credibility intervals (confidence intervals for the DTTL-ML) of point estimates did not include zero with the exception of the covariances marked with an asterisk (*) which had *p* > .05 in the DTTL-ML.

## Data Availability

The data for this study are available at https://osf.io/a9qnc (accessed on 20 July 2024).

## Supplementary Materials

The following supporting information can be downloaded at: https://www.mdpi.com/article/10.3390/jintelligence12100095/s1, Supplementary Material: supplementary_material.ZIP.

## Appendix A. Variance Decomposition in the Divergent Thinking Two-Level Model (DTTL)

The complete variances for any standard indicator (*i* = 1) and any non-standard indicator (*i* ≠ 1) are, respectively, given by

(A1) $$\sigma_{Y_{rt1j}}^{2}=\sigma_{T_{tj}}^{2}+\sigma_{UM_{rtj}}^{2}+\sigma_{\varepsilon_{rt1j}}^{2}$$

and

(A2) $$\sigma_{Y_{rtij}}^{2}={\left(\lambda_{ij}^{T_{j}}\right)}^{2}\sigma_{T_{tj}}^{2}+{\left(\lambda_{ij}^{UM_{j}}\right)}^{2}\sigma_{UM_{rtj}}^{2}+\sigma_{OM_{tij}}^{2}+\sigma_{\varepsilon_{rtij}}^{2},$$

with the variance of the respective true score encapsulating all components except for measurement error (residual):

(A3) $$\sigma_{\tau_{rt1j}}^{2}=\sigma_{T_{tj}}^{2}+\sigma_{UM_{rtj}}^{2}=\sigma_{Y_{rt1j}}^{2}-\sigma_{\varepsilon_{rt1j}}^{2}$$

and

(A4) $$\sigma_{\tau_{rtij}}^{2}={\left(\lambda_{ij}^{T_{j}}\right)}^{2}\sigma_{T_{tj}}^{2}+{\left(\lambda_{ij}^{UM_{j}}\right)}^{2}\sigma_{UM_{rtj}}^{2}+\sigma_{OM_{tij}}^{2}=\sigma_{Y_{rtij}}^{2}-\sigma_{\varepsilon_{rtij}}^{2}.$$

For the true scores of standard indicators, we can define the following meaningful relative variance parameters: The model-implied ICC ($MIICC_{1j}$) depicts the proportion of true score variance that is attributable to variability in the targets:

(A5) $$MIICC_{1j}=\frac{\sigma_{T_{tj}}^{2}}{\sigma_{T_{tj}}^{2}+\sigma_{UM_{rtj}}^{2}}.$$

It can be interpreted as convergent validity form an MTMM-perspective. The unique method specificity coefficient ($UMS_{1j}$) depicts the proportion of true score variance that is attributable to variability in rater-related method-effects:

(A6) $$UMS_{1j}=\frac{\sigma_{UM_{rtj}}^{2}}{\sigma_{T_{tj}}^{2}+\sigma_{UM_{rtj}}^{2}}=1-MIICC_{1j}.$$

For the non-standard indicators, the following meaningful relative variance parameters can be defined: The level-2 (target-level) consistency coefficient ($L2Con_{ij}$) shows the proportion of target-variability in a non-standard indicator (non-standard object) that can be explained by the target-variability of the standard indicator (standard object):

(A7) $$L2Con_{ij}=\frac{{\left(\lambda_{ij}^{T_{j}}\right)}^{2}\sigma_{T_{tj}}^{2}}{{\left(\lambda_{ij}^{T_{j}}\right)}^{2}\sigma_{T_{tj}}^{2}+\sigma_{OM_{tij}}^{2}}.$$

In contrast to this, the level-2 object-method specificity coefficient ($L2OMS_{ij}$) depicts the remaining unexplained proportion of target-level variance that is attributable to object method-effects:

(A8) $$L2OMS_{ij}=\frac{\sigma_{OM_{tij}}^{2}}{{\left(\lambda_{ij}^{T_{j}}\right)}^{2}\sigma_{T_{tj}}^{2}+\sigma_{OM_{tij}}^{2}}=1-L2Con_{ij}.$$

Standard object consistency and non-standard object specificity can also be computed for the overall true score variability, giving the level-1 consistency coefficient ($L1Con_{ij}$) and the level-1 object-method specificity coefficient ($L1OMS_{ij}$), respectively:

(A9) $$L1Con_{ij}=\frac{{\left(\lambda_{ij}^{T_{j}}\right)}^{2}\sigma_{T_{tj}}^{2}}{\sigma_{\tau_{rtij}}^{2}}$$

and

(A10) $$L1OMS_{ij}=\frac{\sigma_{OM_{tij}}^{2}}{\sigma_{\tau_{rtij}}^{2}}$$

The unique method specificity coefficient may also be defined for the non-standard indicators:

(A11) $$UMS_{ij}=\frac{{\left(\lambda_{ij}^{UM_{j}}\right)}^{2}\sigma_{UM_{rtj}}^{2}}{\sigma_{\tau_{rtij}}^{2}}.$$

Lastly, the model-implied ICC for non-standard indicators must respect both the target-specific trait-variance and the target-specific object method-effect variance:

(A12) $$MIICC_{ij}=\frac{{\left(\lambda_{ij}^{T_{j}}\right)}^{2}\sigma_{T_{tj}}^{2}+\sigma_{OM_{tij}}^{2}}{\sigma_{\tau_{rtij}}^{2}}=1-UMS_{ij}.$$

For all indicators, regardless of whether they are standard indicators or not, we can, of course, compute reliability ($REL_{ij}$) as the proportion of true score variance in the complete variance:

(A13) $$REL_{ij}=\frac{\sigma_{\tau_{rtij}}^{2}}{\sigma_{Y_{rtij}}^{2}}=1-\frac{\sigma_{\varepsilon_{rtij}}^{2}}{\sigma_{Y_{rtij}}^{2}}.$$

## Appendix B. Prior-Specifications within the DTCC and the DTTL-B of the Presented Application

For our selection of prior-distributions, we followed the rationale put forward by Koch et al. (2016) for cross-classified CTC(M − 1) models. Within the DTCC, for all residual variances and variances of latent variables that are uncorrelated with any other latent variables, we set uninformative priors following an inverse gamma distribution:

$$\begin{array}{l} \sigma_{R_{rj}}^{2}\sim \Gamma^{-1}(0.001,0.001),\\ \sigma_{INT_{rtj}}^{2}\sim \Gamma^{-1}(0.001,0.001),\\ \sigma_{\varepsilon_{rtij}}^{2}\sim \Gamma^{-1}(0.001,0.001). \end{array}$$

For all blocks of covarying latent variables (the block of the two latent trait variables and the block of the latent object method-effect variables), we set uninformative priors following an inverse Wishart distribution:

$$\begin{array}{l} \left(\begin{array}{cc} \sigma_{T_{t1}}^{2} & \sigma_{T_{1}T_{2}}\\ & \sigma_{T_{t2}}^{2} \end{array}\right)\sim Wishart^{-1}(I,2),\\ \left(\begin{array}{cccc} \sigma_{OM_{t21}}^{2} & \sigma_{OM_{21}OM_{31}} & \sigma_{OM_{21}OM_{22}} & \sigma_{OM_{21}OM_{32}}\\ & \sigma_{OM_{t31}}^{2} & \sigma_{OM_{31}OM_{22}} & \sigma_{OM_{31}OM_{32}}\\ & & \sigma_{OM_{t22}}^{2} & \sigma_{OM_{22}OM_{32}}\\ & & & \sigma_{OM_{t32}}^{2} \end{array}\right)\sim Wishart^{-1}(I,2). \end{array}$$

For intercepts and factor-loadings, we used informative priors. Since the intercepts merely reflect unconditional expectations of observed variables, they should be estimated close to their means. Accordingly, we used normal distributions with the indicator means and low variance:

$$\begin{array}{l} \mu_{11}\sim N(2.99,0.1),\\ \mu_{21}\sim N(3.07,0.1),\\ \mu_{31}\sim N(3.03,0.1),\\ \mu_{12}\sim N(2.90,0.1),\\ \mu_{22}\sim N(3.13,0.1),\\ \mu_{32}\sim N(2.93,0.1). \end{array}$$

For factor loadings on the rater- and interaction-level, we used normal distributions with an expectancy of one and low variance since one would expect a homogenous measurement. For factor loadings of the latent trait variables, however, we used a normal distribution with a lower expectancy and higher variance because the cross-loadings of traits (measured with the standard method) on non-standard method-indicators are usually lower in CTC(M − 1) models (Eid et al. 2003, 2008; Koch et al. 2016; Nussbeck et al. 2006):

$$\begin{array}{l} \lambda_{ij}^{R_{j}}\sim N(1,0.1),\\ \lambda_{ij}^{INT_{j}}\sim N(1,0.1),\\ \lambda_{ij}^{T_{j}}\sim N(0.7,0.2). \end{array}$$

Within the DTTL-B, we maintained all priors for intercepts and parameters on the target-level (level-2) and used the priors of the DTCC’s interaction-level for level-1 of the DTTL-B, that is:

$$\begin{array}{l} \sigma_{UM_{rtj}}^{2}\sim \Gamma^{-1}(0.001,0.001),\\ \sigma_{\varepsilon_{rtij}}^{2}\sim \Gamma^{-1}(0.001,0.001),\\ \lambda_{ij}^{UM_{j}}\sim N(1,0.1). \end{array}$$
