# Supplementary material for: Decomposing the True Score Variance in Rated Responses to Divergent Thinking-Tasks for Assessing Creativity: A Multitrait–Multimethod Analysis
Source: J Intell. 2024 Sep 27;12(10):95. doi: 10.3390/jintelligence12100095 (PMC11508196; doi:10.3390/jintelligence12100095)
Supplement: Supplementary file 1 [file jintelligence-12-00095-s001.zip › supplementary_material/comparison_GR_FIXED.pdf]

Supplementary Material for the paper "Decomposing the True Score Variance in Rated Responses to Divergent Thinking-Tasks for Assessing Creativity: A Multitrait-Multimethod Analysis":

Comparison of using the Gelman-Rubin criterion versus using a fixed number of 1,000,000 iterations for estimating the Divergent Thinking Cross-Classified Model

The Divergent Thinking Cross-Classified Model (DTCC) estimated with the Gelman-Rubin criterion of a maximum potential scale reduction (PSR) factor of 1.1 (GR 1.1) yielded good model fit ($M(\Delta\chi^2)$ = -3.845, 95%-COI = [-54.944, 64.395], p = .447). The model fit statistics are very similar to those yielded with a fixed number of 1,000,000 iterations (FIXED) as reported in the main manuscript and repeated here: $M(\Delta\chi^2)$ = -4.451, 95%-COI = [-56.533, 64.945], p = .438. Table S1 compares the parameter estimates and relative variances across the two estimation strategies. Most parameters remained either unaffected by the estimation choice or had only slight changes. The highest difference was observed for the rater-effect variance of the second construct (creative quality). The fixed iterations-approach yielded a point estimate that was by 0.048 lower (FIXED: $\sigma^2_{R_{r2}}$ = 0.328, GR 1.1: $\sigma^2_{R_{r2}}$ = 0.280). Accordingly, convergence for this parameter estimate was rather instable within the DTCC. We conjecture that this is due to the small number of raters (three) used for the creative quality-ratings as no problems occurred for the rater-effect variance of cleverness (which had four raters). In any case, the main conclusions remain unaffected by the selection of the convergence-criterion.

Table S1. Parameter estimates and relative variances for the Divergent Thinking Cross-Classified model (DTCC) using two different Bayesian estimation approaches.

	**FIXED**						**GR 1.1**					
**Parameter**	Y_{11}	Y_{21}	Y_{31}	Y_{12}	Y_{22}	Y_{32}	Y_{11}	Y_{21}	Y_{31}	Y_{12}	Y_{22}	Y_{32}
μ_{ij}	2.993	3.066	3.026	2.907	3.132	2.927	2.960	3.051	3.020	2.968	3.213	3.968
$\lambda_{ij}^{T_j}$	1	0.489	0.498	1	0.540	0.459	1	0.482	0.494	1	0.527	0.457
$\lambda_{ij}^{R_j}$	1	0.715	0.502	1	1.108	0.583	1	0.711	0.502	1	1.111	0.583
$\lambda_{ij}^{INT_j}$	1	0.997	0.908	1	0.932	1.004	1	0.987	0.892	1	0.935	1.001
$\sigma^2_{\varepsilon_{rtij}}$	0.324	0.261	0.273	0.158	0.209	0.188	0.323	0.260	0.275	0.158	0.208	0.187
$\sigma^2_{T_{tj}}$		0.588			0.223			0.589			0.224	
$\sigma^2_{OM_{tij}}$		0.468	0.429		0.210	0.182		0.471	0.424		0.211	0.184
$\sigma^2_{R_{rj}}$		0.055			0.328			0.051			0.280	
$\sigma^2_{INT_{rtj}}$		0.047			0.004			0.048			0.004	
$\sigma_{T_1T_2}$			0.316 (.876)						0.317 (.875)			
$\sigma_{OM_{21}OM_{31}}$			0.187 (.420)						0.189 (.425)			
$\sigma_{OM_{21}OM_{22}}$			0.285 (.912)						0.284 (.905)			
$\sigma_{OM_{21}OM_{32}}$			0.075 (.260)						0.078 (.265)			
$\sigma_{OM_{31}OM_{22}}$			0.073 (.245)						0.070 (.235)			
$\sigma_{OM_{31}OM_{32}}$			0.251 (.901)						0.253 (.906)			
$\sigma_{OM_{22}OM_{32}}$			0.051 (.263)						0.050 (.255)			
$L2Con_{ij}$		.231	.254		.237	.205		.224	.254		.228	.202
$L2OMS_{ij}$		.769	.746		.763	.795		.776	.746		.772	.798
$L1Con_{ij}$		.201	.228		.091	.126		.196	.229		.092	.127
$L1OMS_{ij}$		.672	.674		.306	.520		.683	.676		.336	.548
$MIICC_{ij}$	.846	.885	.911	.401	.405	.663	.849	.888	.913	.439	.438	.696
RMS_{ij}	.080	.041	.022	.590	.589	.321	.074	.037	.020	.550	.554	.285
IMS_{ij}	.065	.065	.060	.006	.004	.009	.067	.066	.060	.007	.005	.010
UMS_{ij}	.154	.115	.089	.599	.595	.337	.151	.112	.087	.561	.562	.304
REL_{ij}	.688	.730	.701	.780	.767	.655	.687	.728	.697	.767	.752	.647

Notes. N = 202. FIXED = Bayesian estimation using a fixed number of 1,000,000 iterations (highest PSR factor at the last iteration = 1.021), GR 1.1 = Bayesian estimation with the Gelman-Rubin criterion (highest acceptable PSR factor = 1.1), Y_{ij} = AUT-score variable of object i (1 = rope, 2 = garbage-bag, 3 = paperclip) scored for construct j (1 = cleverness, 2 = creative quality). μ indicates an intercept, λ indicates a factor-loading, σ^2 indicates a variance, and σ indicates a covariance. ε_{rtij} = residual of an AUT-score variable, T_{tj} = latent trait variable for construct j, OM_{tij} = (DT-) object-specific method-effect variable for non-reference object i for construct j, R_{rj} = rater-effect variable for construct j, INT_{rtj} = interaction-effect variable for construct j, $L2Con_{ij}$ = level-2 consistency for non-reference object i of construct j, $L2OMS_{ij}$ = level-2 object-method specificity for non-reference object i of construct j, $L1Con_{ij}$ = level-1 consistency for non-reference object i of construct j, $L1OMS_{ij}$ = level-1 object-method specificity for non-reference object i of construct j, $MIICC_{ij}$ = model-implied intra-class correlation of indicator i for construct j, RMS_{ij} = rater specificity of indicator i for construct j, IMS_{ij} = interaction specificity of indicator i for construct j, UMS_{ij} = unique method specificity of indicator i for construct j, REL_{ij} = reliability of indicator i for construct j. Values of 1 were fixed. Numbers in parentheses depict correlations. Two-sided 95%-credibility intervals of all point estimates did not include zero.
