# Supplementary material for: Decomposing the True Score Variance in Rated Responses to Divergent Thinking-Tasks for Assessing Creativity: A Multitrait–Multimethod Analysis
Source: J Intell. 2024 Sep 27;12(10):95. doi: 10.3390/jintelligence12100095 (PMC11508196; doi:10.3390/jintelligence12100095)

Supplementary Material for the paper "Decomposing the True Score Variance in Rated Responses to Divergent Thinking-Tasks for Assessing Creativity: A Multitrait-Multimethod Analysis":

Trace Plots for Bayesian Analyses

This Document shows the trace plots for the parameters of the Divergent Thinking Cross-Classified Model (DTCC) and for the parameters of the Divergent Thinking Two-Level Model-B (DTTL-B) both estimated using Bayesian Markov Chain Monte Carlo simulation with the standard Gelman-Rubin criterion (highest acceptable potential scale reduction factor = 1.1). The plots shown on pages 2 – 10 refer to the DTCC; the plots shown on pages 11 – 18 refer to the DTTL-B. The parameter for each plot is depicted in the upper right corner of the plot. The same parameter labels as in the main manuscript are used (see, for example, Figure 2 and Table 2). Each plot is structured in the same way. The x-axis depicts the iterations, the y-axis depicts the parameter-space. The vertical red line in each plot marks the end of the first half of all iterations. Posterior parameter distributions are based on the second half of all iterations (the first half was treated as burn-in). Three chains were used, each represented by a differently colored trace-line (blue, green, and red).

DTCC

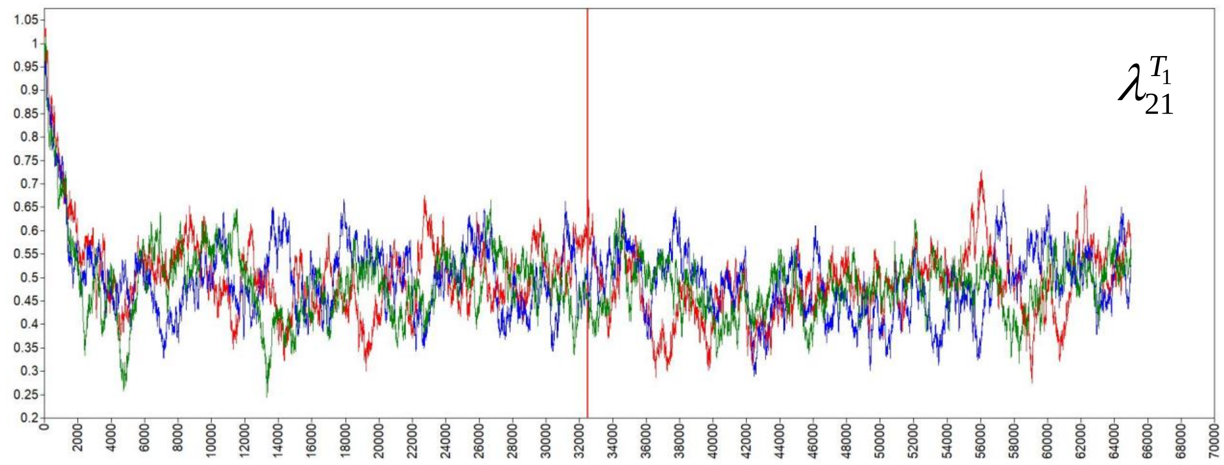

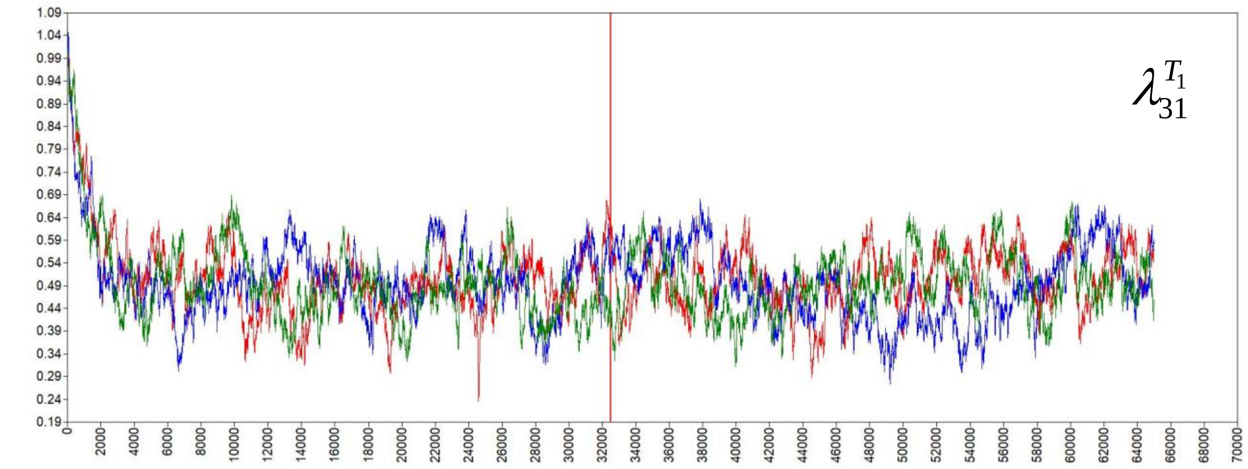

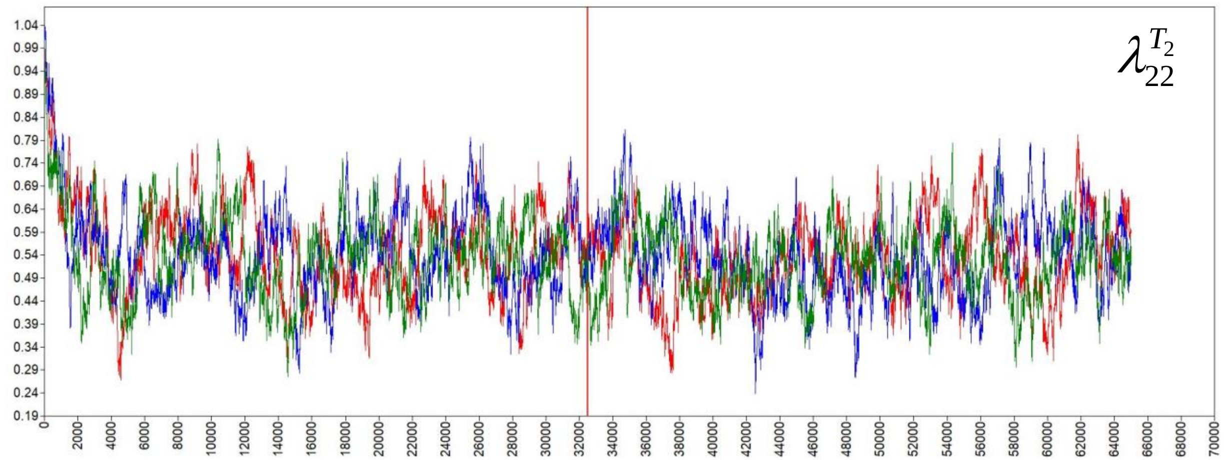

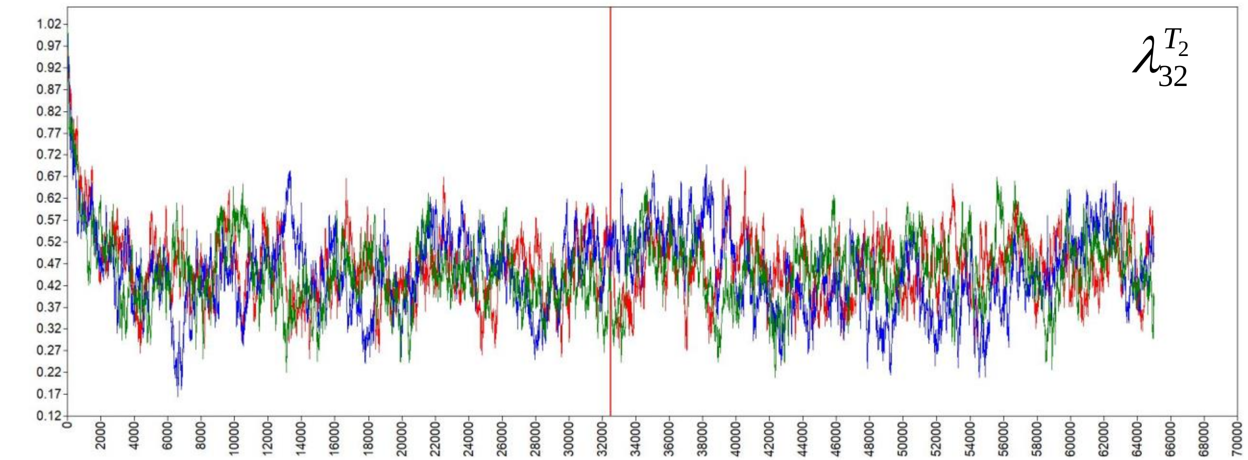

DTCC

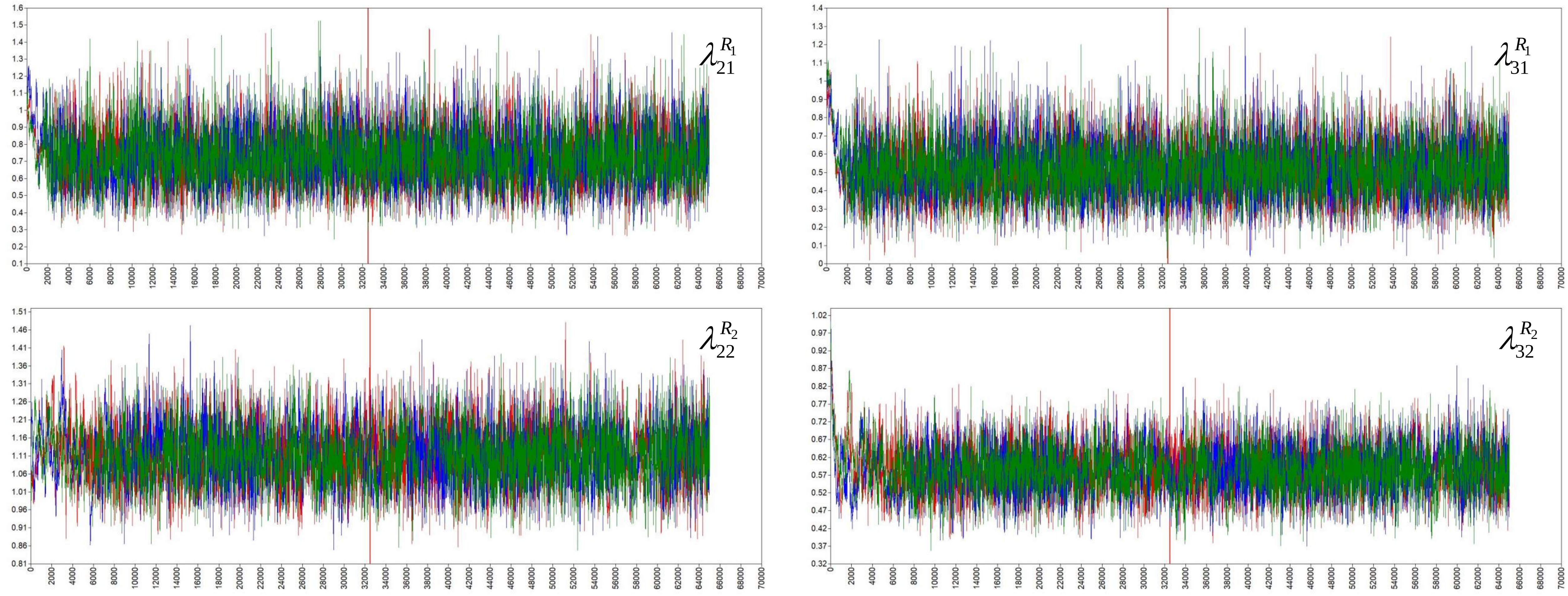

DTCC

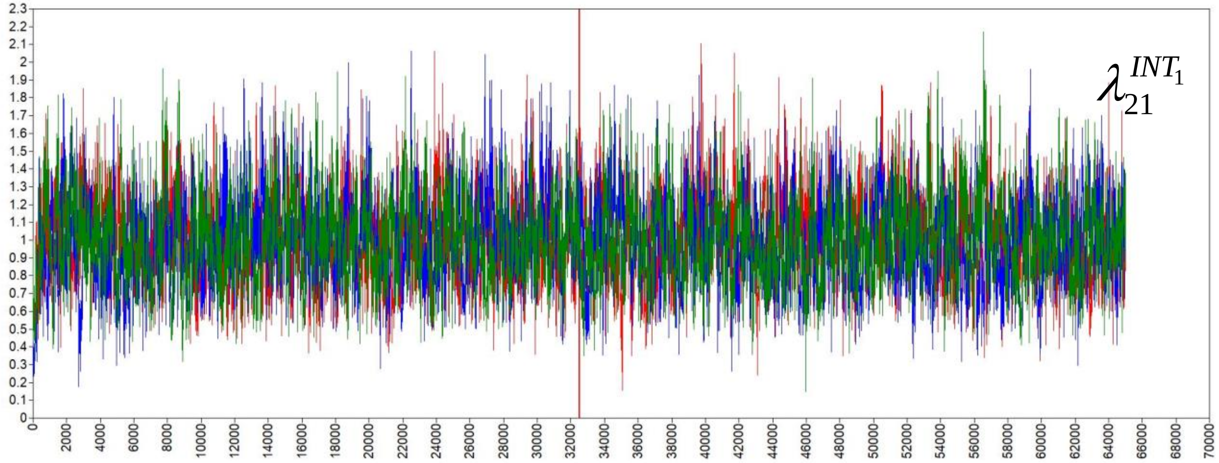

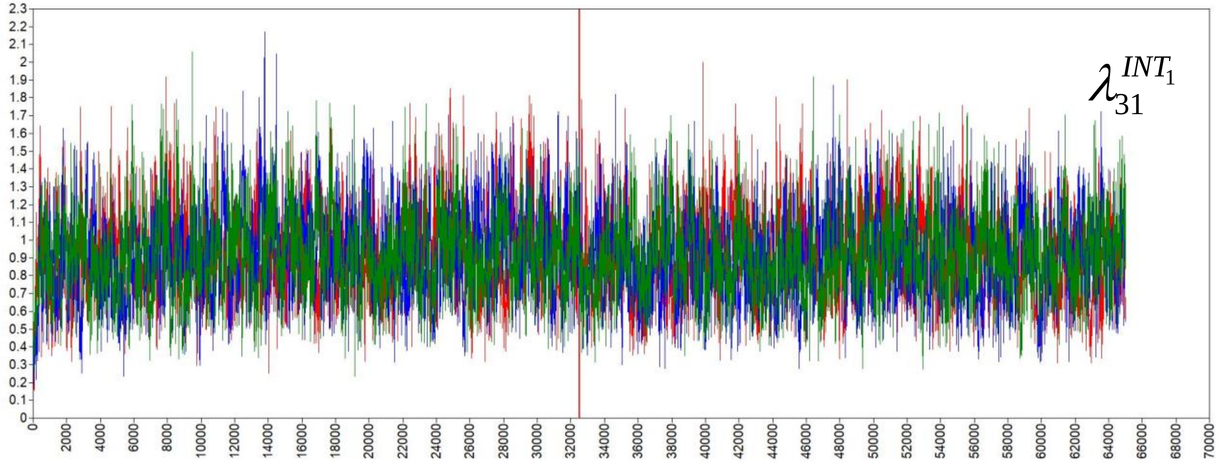

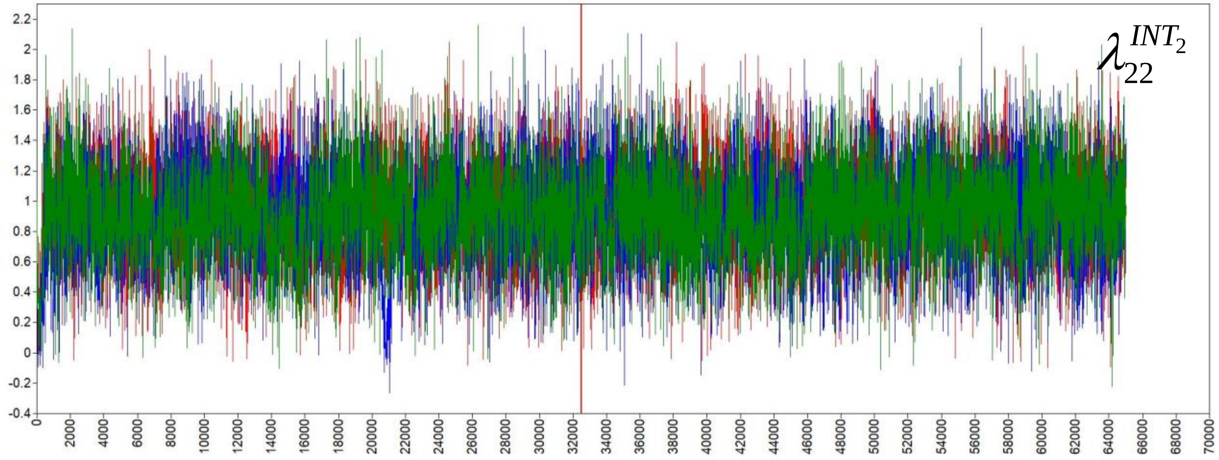

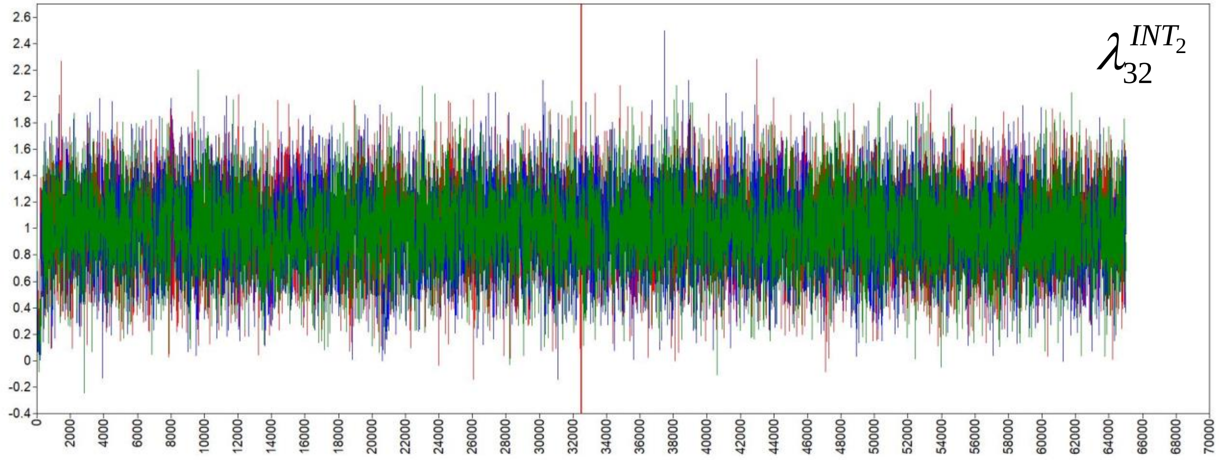

DTCC

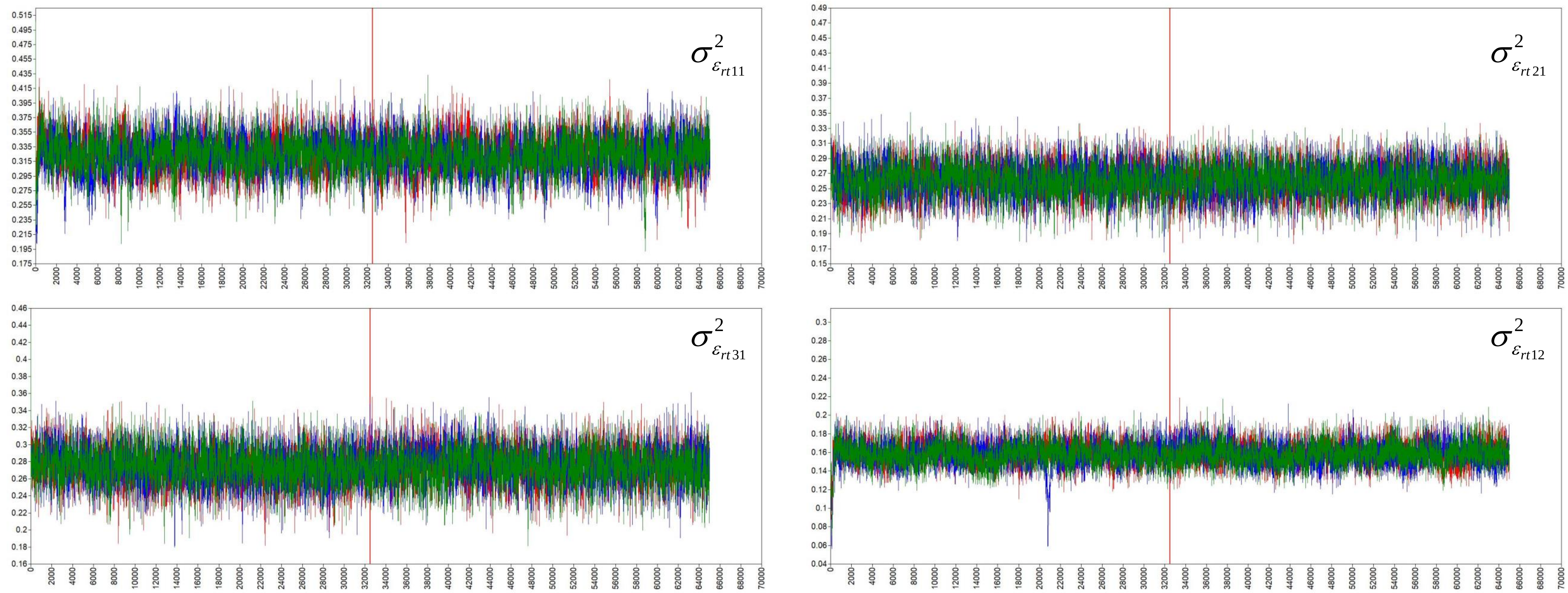
$\sigma^2_{\varepsilon_{rt11}}$
$\sigma^2_{\varepsilon_{rt21}}$
$\sigma^2_{\varepsilon_{rt31}}$
$\sigma^2_{\varepsilon_{rt12}}$

DTCC

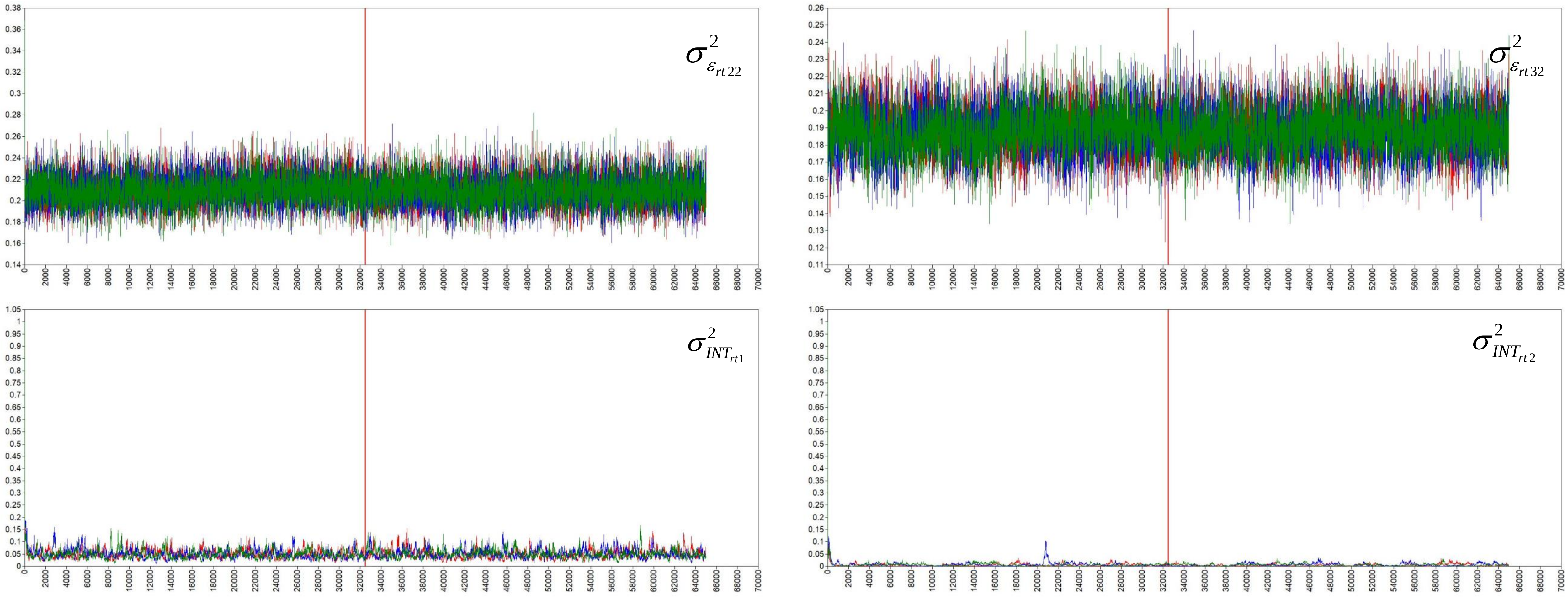
$\sigma^2_{\varepsilon_{rt22}}$
$\sigma^2_{\varepsilon_{rt32}}$
$\sigma^2_{INT_{rt1}}$
$\sigma^2_{INT_{rt2}}$

DTCC

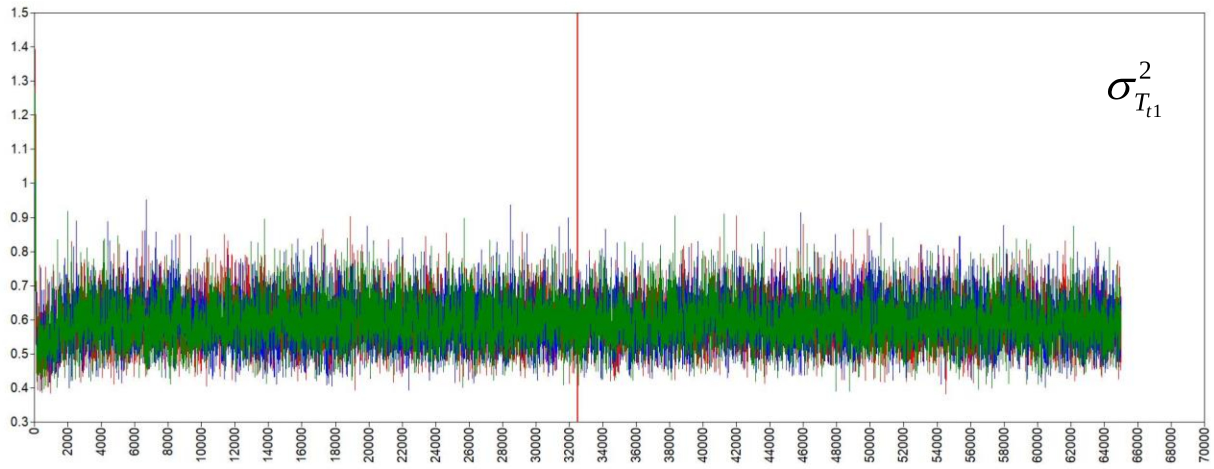

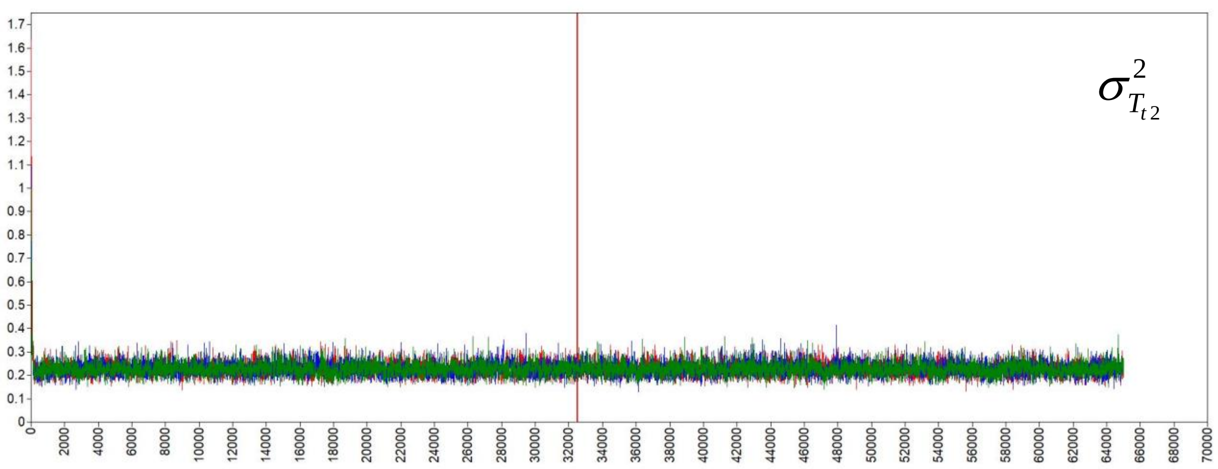

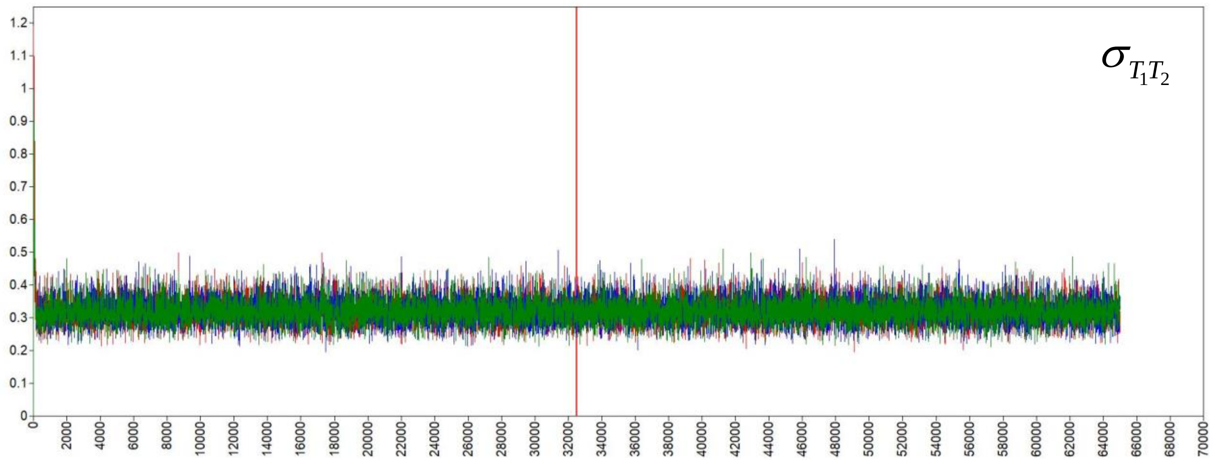

DTCC

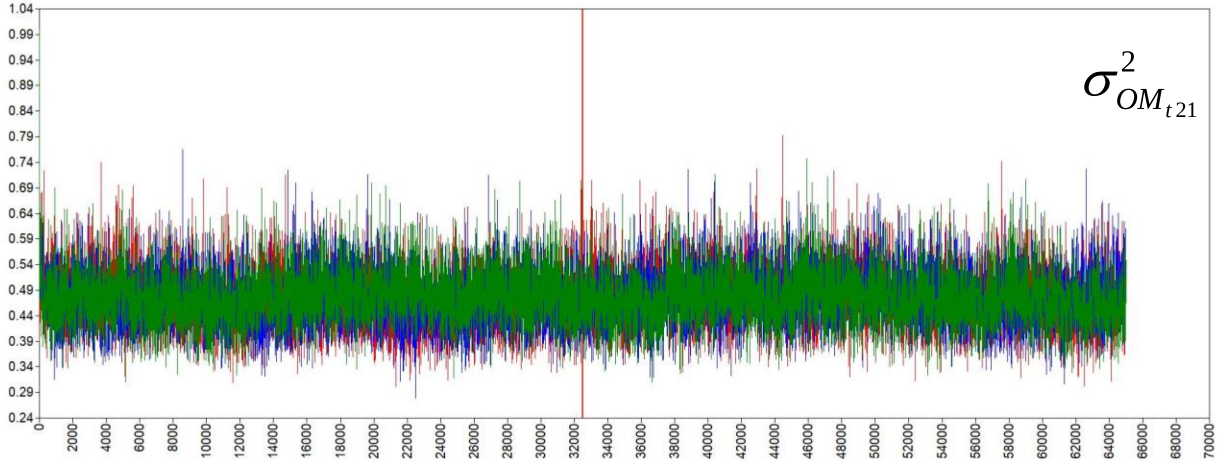

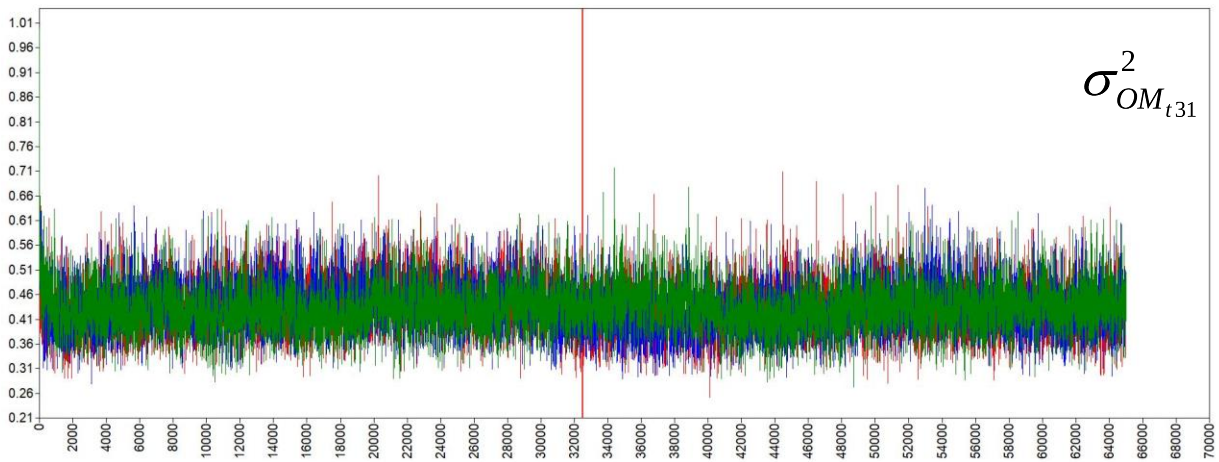

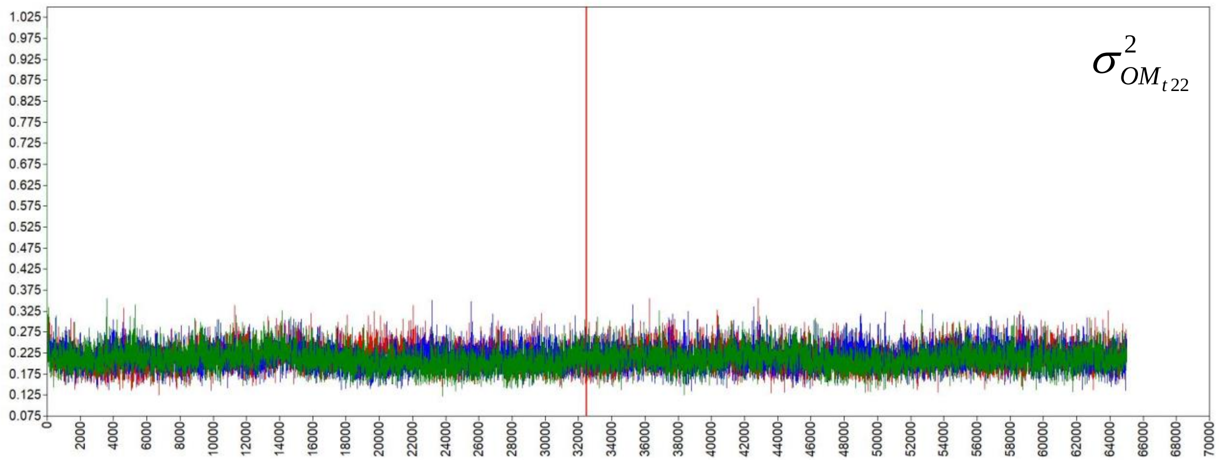

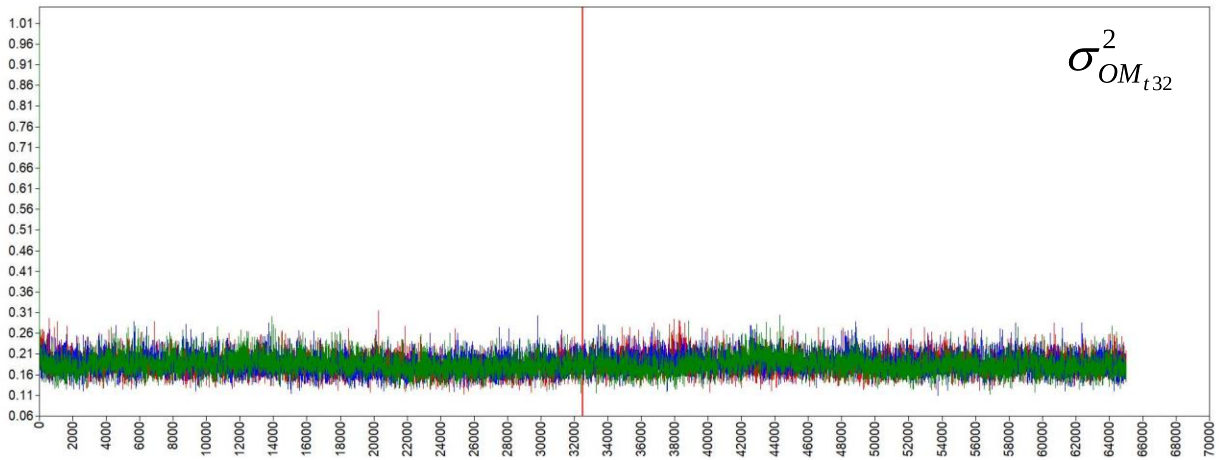

DTCC

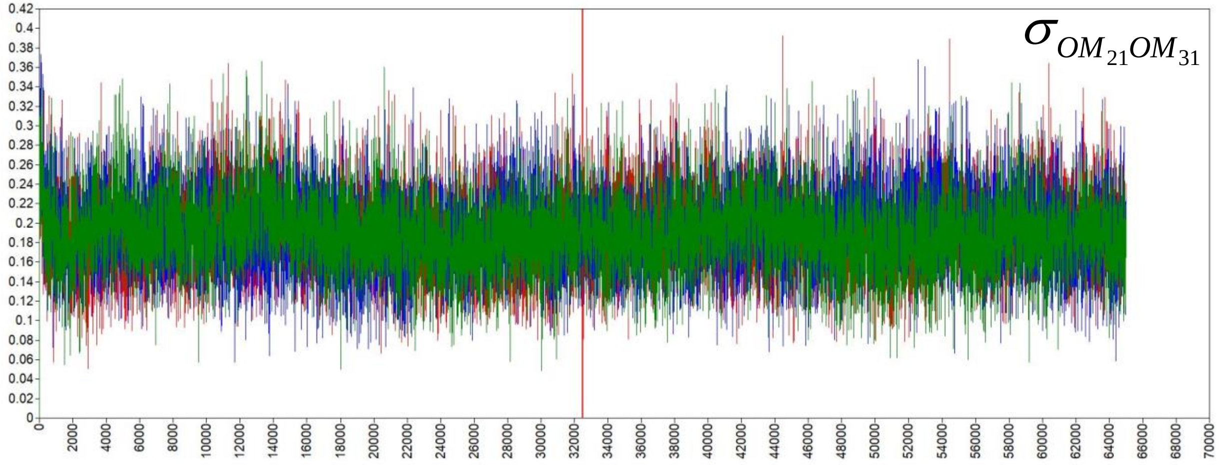
$\sigma_{OM_{21}OM_{31}}$

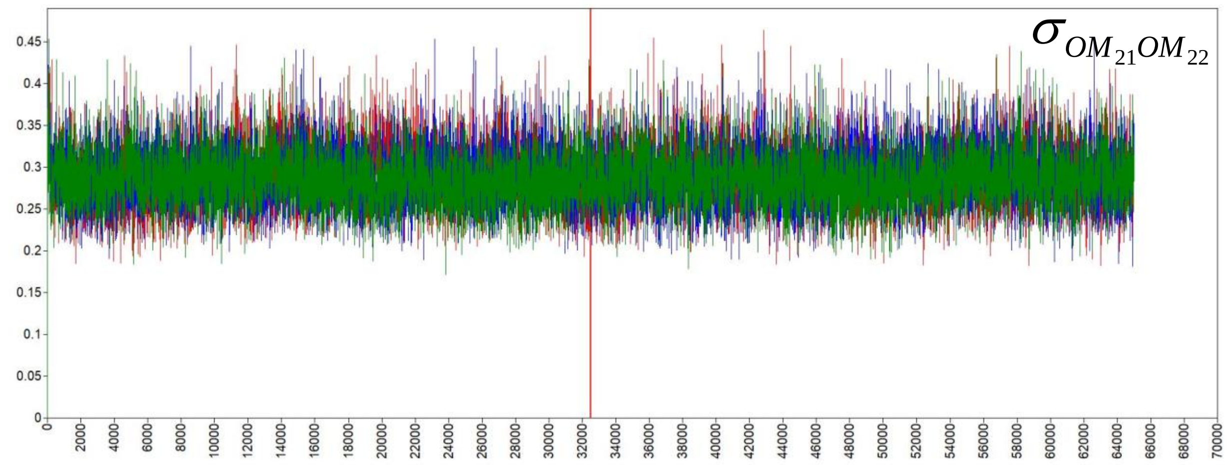
$\sigma_{OM_{21}OM_{22}}$

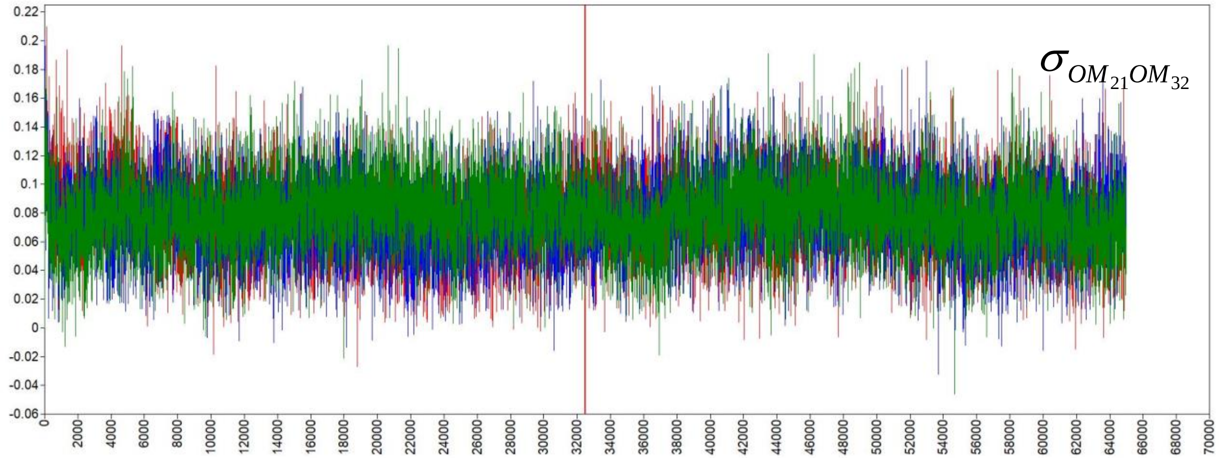
$\sigma_{OM_{21}OM_{32}}$

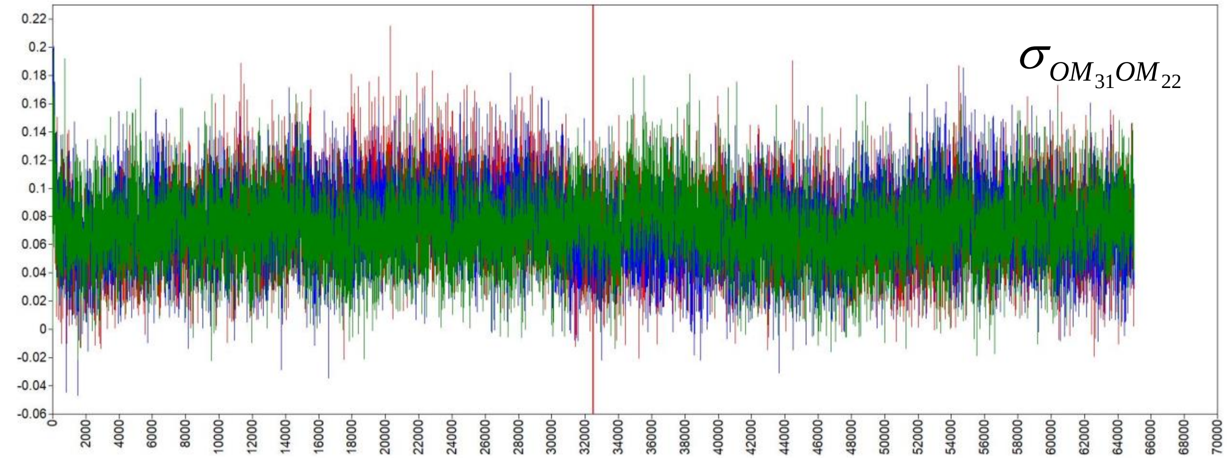
$\sigma_{OM_{31}OM_{22}}$

DTCC

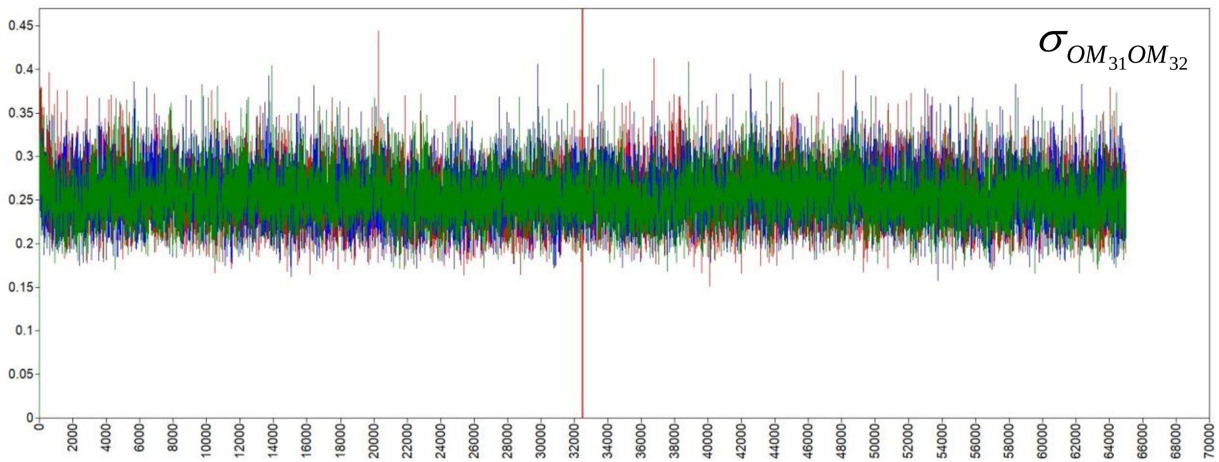

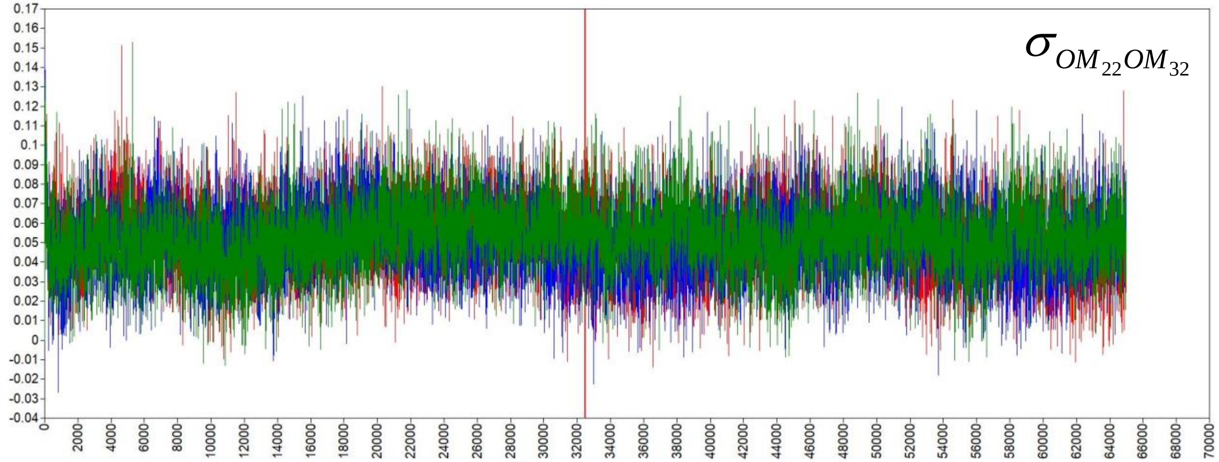

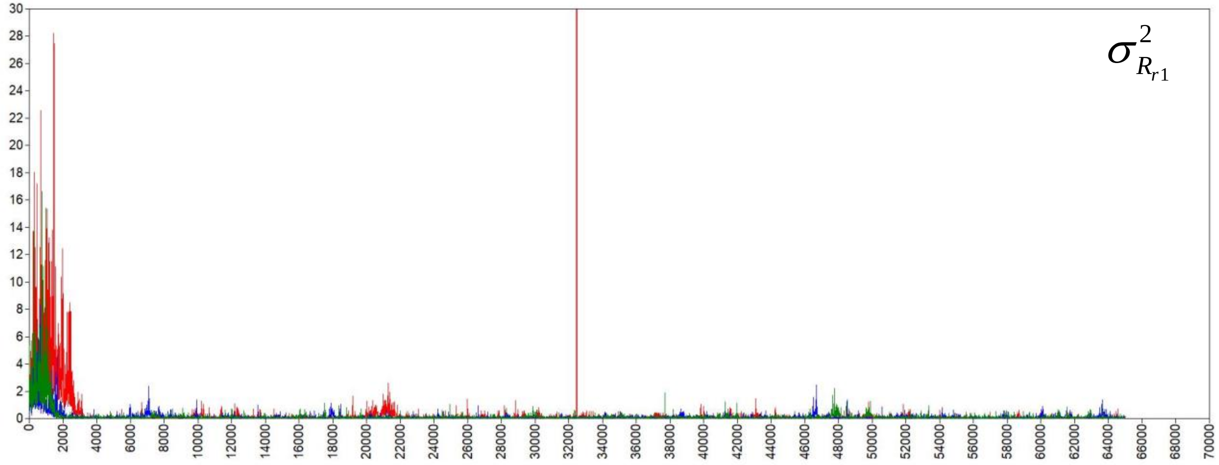

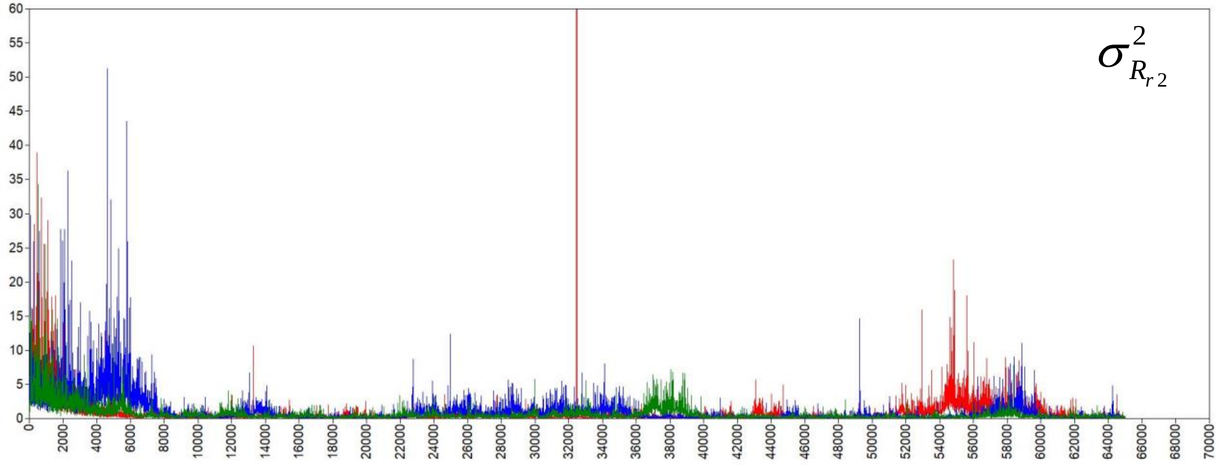

DTTL-B

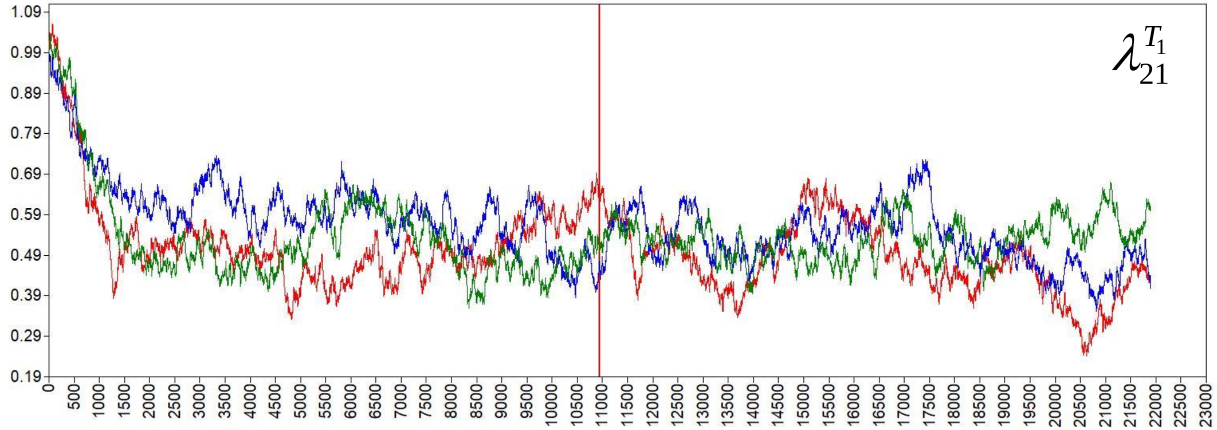
$\lambda_{21}^{T_1}$
1.09 0.99 0.89 0.79 0.69 0.59 0.49 0.39 0.29 0.19
0 500 1000 1500 2000 2500 3000 3500 4000 4500 5000 5500 6000 6500 7000 7500 8000 8500 9000 9500 10000 10500 11000 11500 12000 12500 13000 13500 14000 14500 15000 15500 16000 16500 17000 17500 18000 18500 19000 19500 20000 20500 21000 21500 22000 22500 23000

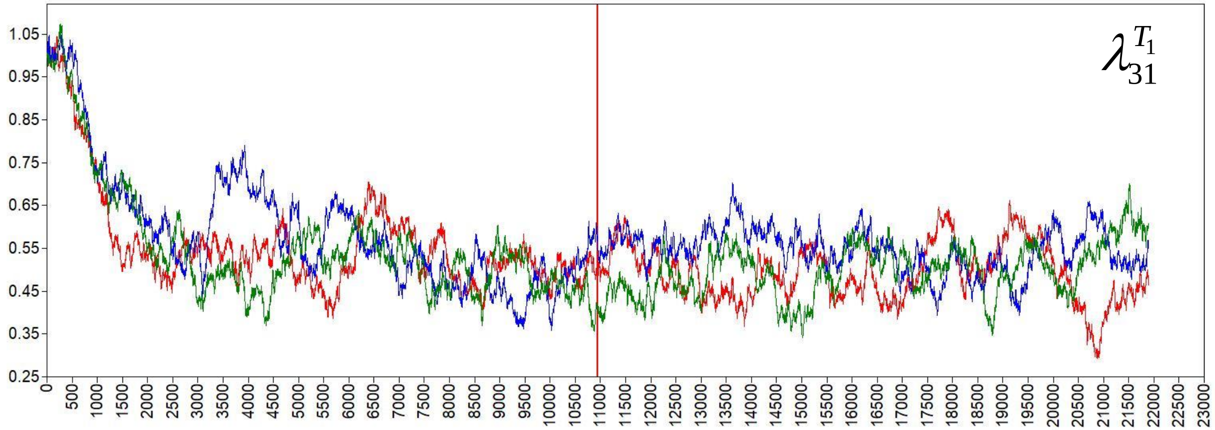
$\lambda_{31}^{T_1}$
1.05 0.95 0.85 0.75 0.65 0.55 0.45 0.35 0.25
0 500 1000 1500 2000 2500 3000 3500 4000 4500 5000 5500 6000 6500 7000 7500 8000 8500 9000 9500 10000 10500 11000 11500 12000 12500 13000 13500 14000 14500 15000 15500 16000 16500 17000 17500 18000 18500 19000 19500 20000 20500 21000 21500 22000 22500 23000

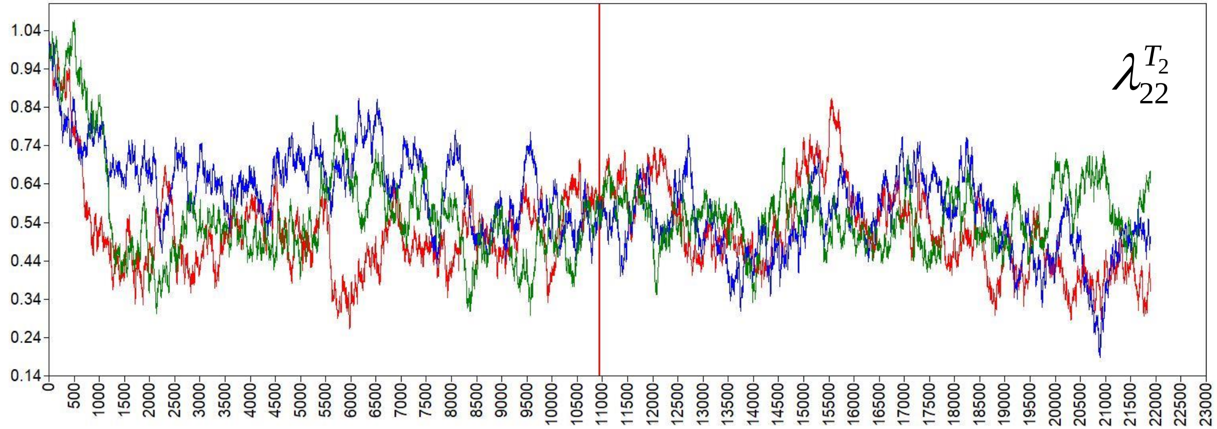
$\lambda_{22}^{T_2}$
1.04 0.94 0.84 0.74 0.64 0.54 0.44 0.34 0.24 0.14
0 500 1000 1500 2000 2500 3000 3500 4000 4500 5000 5500 6000 6500 7000 7500 8000 8500 9000 9500 10000 10500 11000 11500 12000 12500 13000 13500 14000 14500 15000 15500 16000 16500 17000 17500 18000 18500 19000 19500 20000 20500 21000 21500 22000 22500 23000

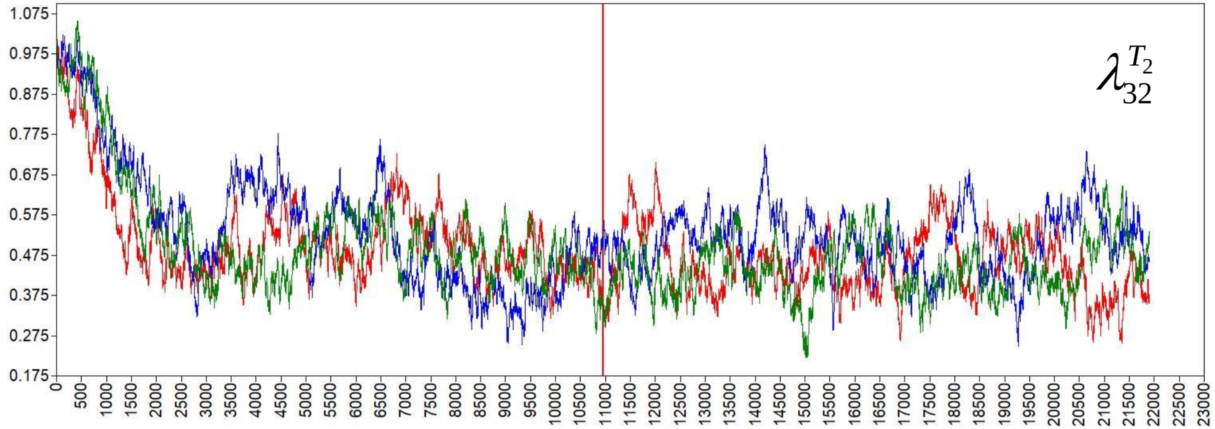
$\lambda_{32}^{T_2}$
1.075 0.975 0.875 0.775 0.675 0.575 0.475 0.375 0.275 0.175
0 500 1000 1500 2000 2500 3000 3500 4000 4500 5000 5500 6000 6500 7000 7500 8000 8500 9000 9500 10000 10500 11000 11500 12000 12500 13000 13500 14000 14500 15000 15500 16000 16500 17000 17500 18000 18500 19000 19500 20000 20500 21000 21500 22000 22500 23000

DTTL-B

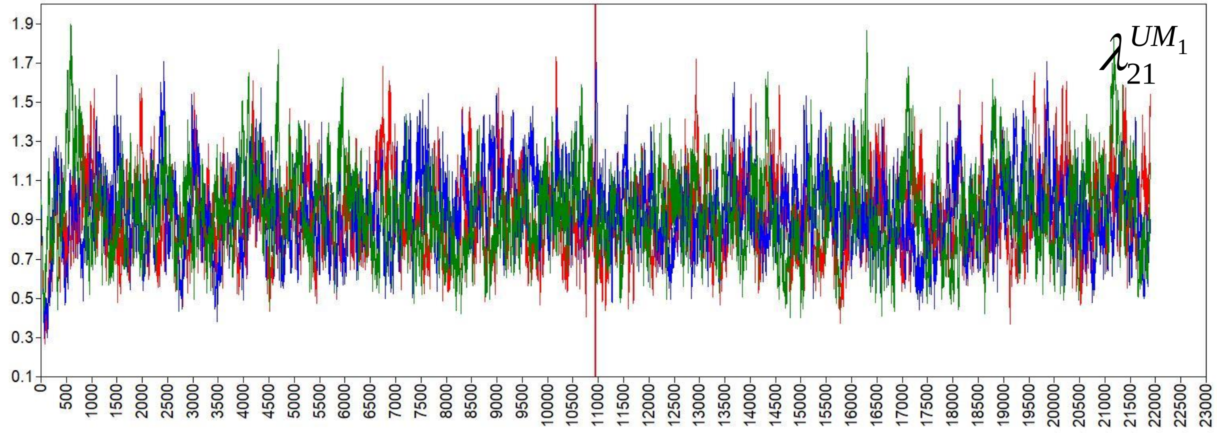

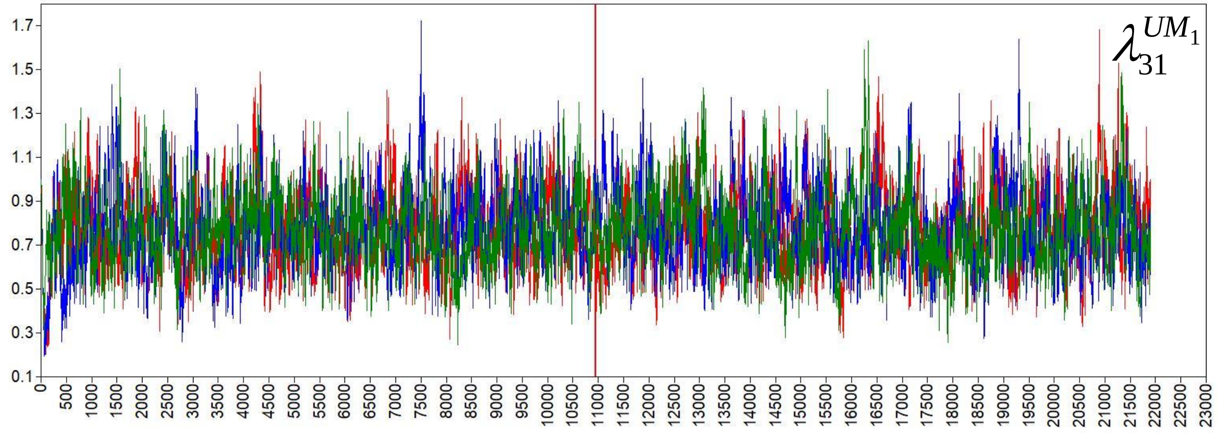

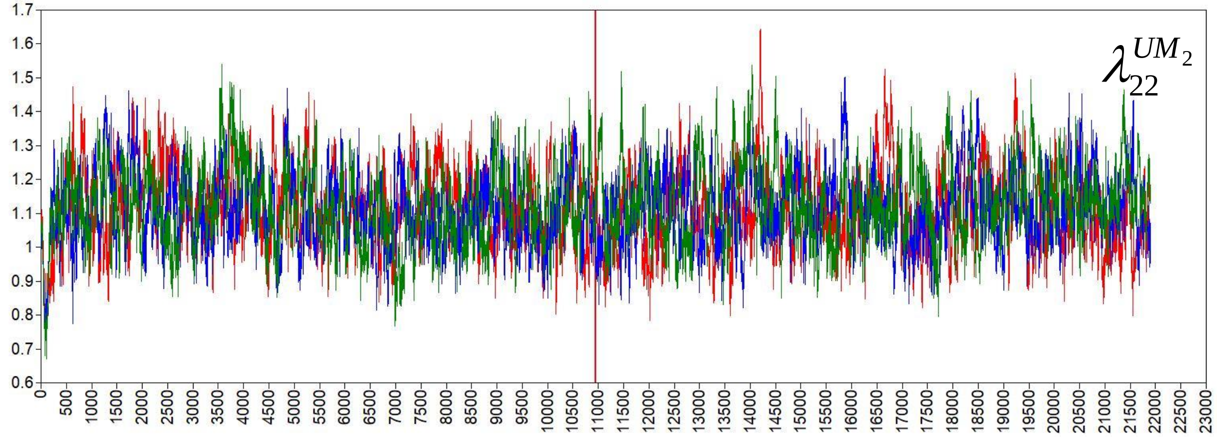

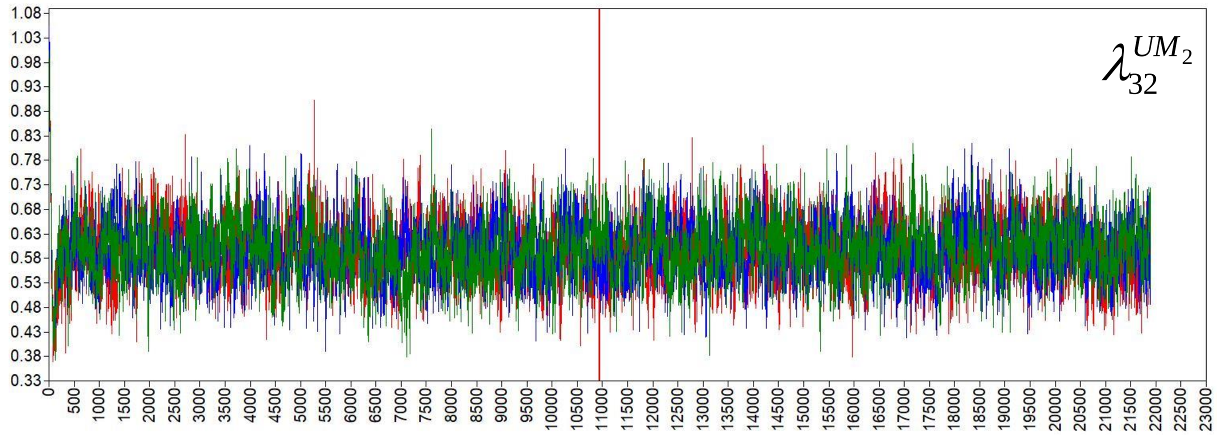

DTTL-B

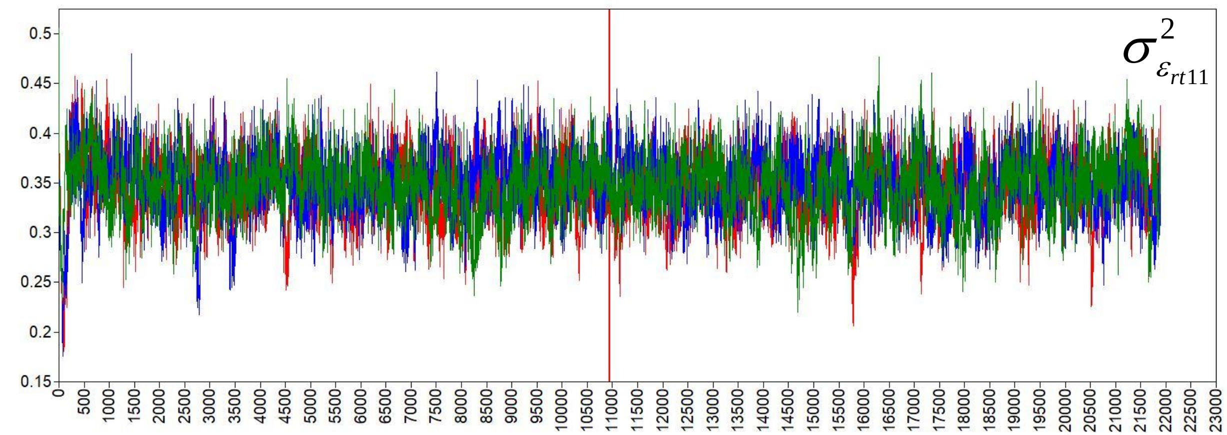
$\sigma^2_{\varepsilon_{rt11}}$
0.5 0.45 0.4 0.35 0.3 0.25 0.2 0.15
0 500 1000 1500 2000 2500 3000 3500 4000 4500 5000 5500 6000 6500 7000 7500 8000 8500 9000 9500 10000 10500 11000 11500 12000 12500 13000 13500 14000 14500 15000 15500 16000 16500 17000 17500 18000 18500 19000 19500 20000 20500 21000 21500 22000 22500 23000

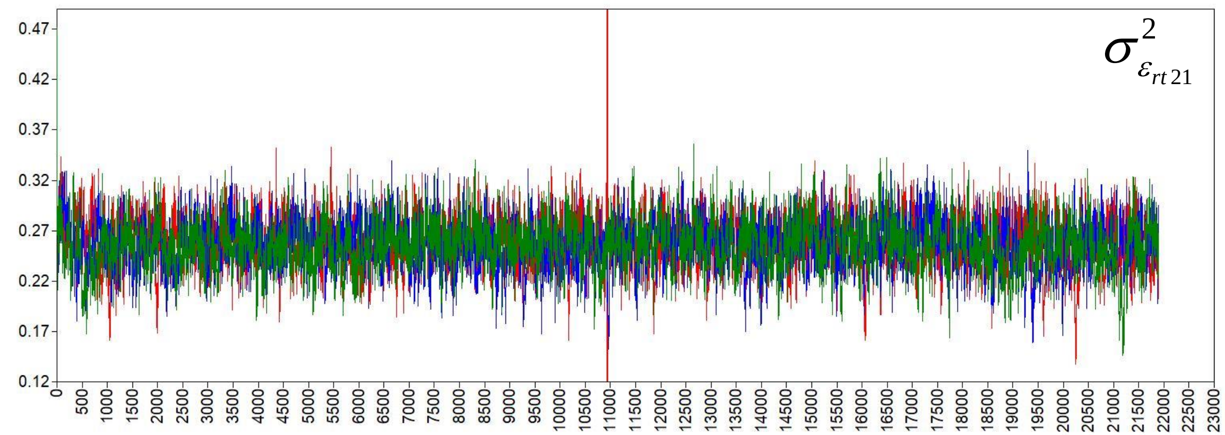
$\sigma^2_{\varepsilon_{rt21}}$
0.47 0.42 0.37 0.32 0.27 0.22 0.17 0.12
0 500 1000 1500 2000 2500 3000 3500 4000 4500 5000 5500 6000 6500 7000 7500 8000 8500 9000 9500 10000 10500 11000 11500 12000 12500 13000 13500 14000 14500 15000 15500 16000 16500 17000 17500 18000 18500 19000 19500 20000 20500 21000 21500 22000 22500 23000

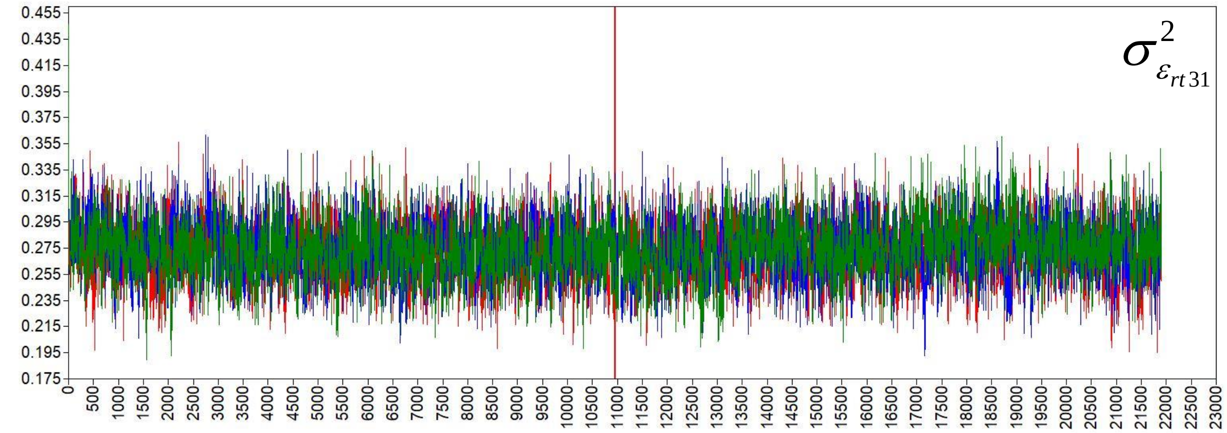
$\sigma^2_{\varepsilon_{rt31}}$
0.455 0.435 0.415 0.395 0.375 0.355 0.335 0.315 0.295 0.275 0.255 0.235 0.215 0.195 0.175
0 500 1000 1500 2000 2500 3000 3500 4000 4500 5000 5500 6000 6500 7000 7500 8000 8500 9000 9500 10000 10500 11000 11500 12000 12500 13000 13500 14000 14500 15000 15500 16000 16500 17000 17500 18000 18500 19000 19500 20000 20500 21000 21500 22000 22500 23000

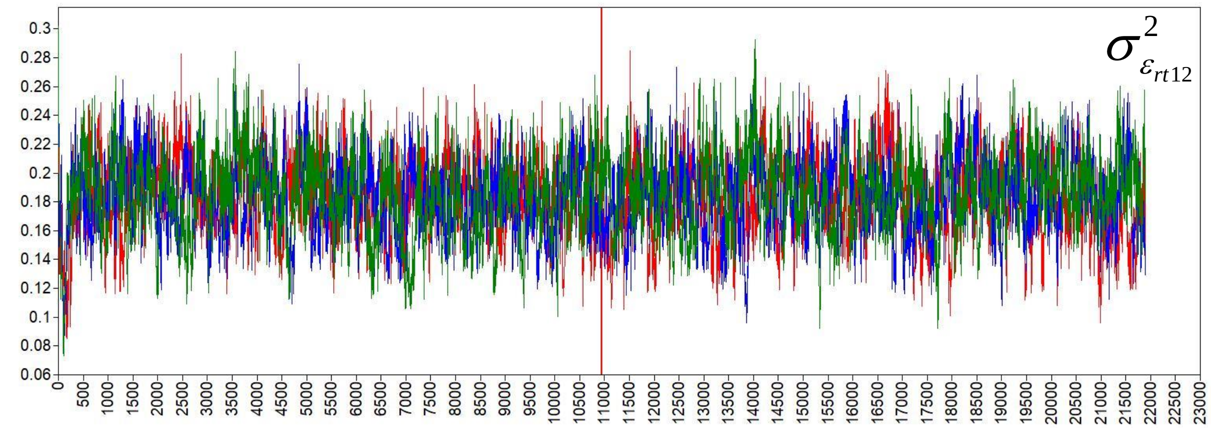
$\sigma^2_{\varepsilon_{rt12}}$
0.3 0.28 0.26 0.24 0.22 0.2 0.18 0.16 0.14 0.12 0.1 0.08 0.06
0 500 1000 1500 2000 2500 3000 3500 4000 4500 5000 5500 6000 6500 7000 7500 8000 8500 9000 9500 10000 10500 11000 11500 12000 12500 13000 13500 14000 14500 15000 15500 16000 16500 17000 17500 18000 18500 19000 19500 20000 20500 21000 21500 22000 22500 23000

DTTL-B

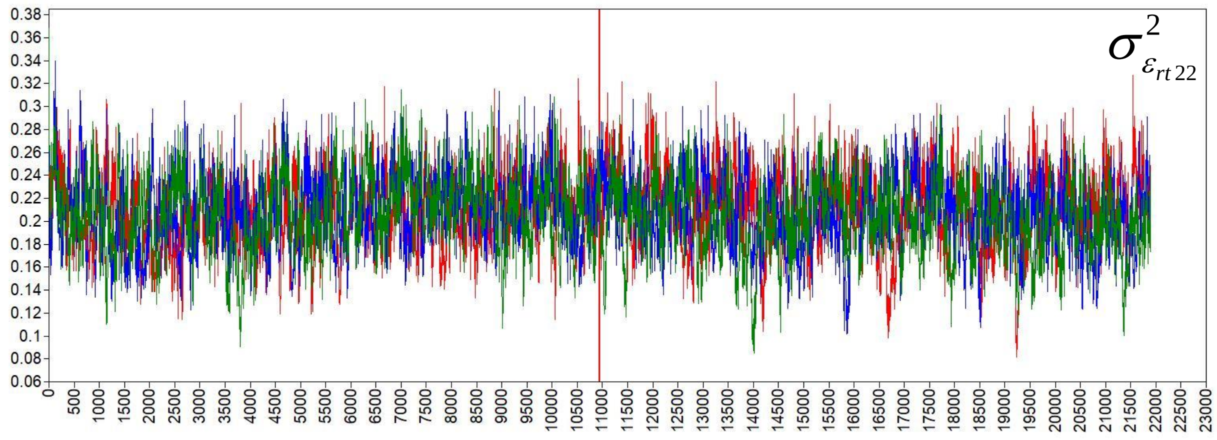
$\sigma^2_{\varepsilon_{rt\,22}}$

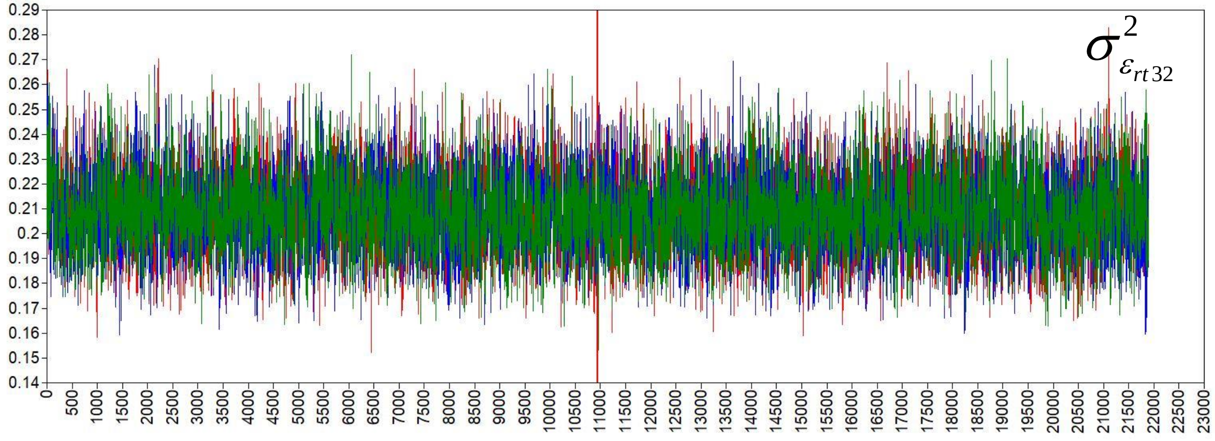
$\sigma^2_{\varepsilon_{rt\,32}}$

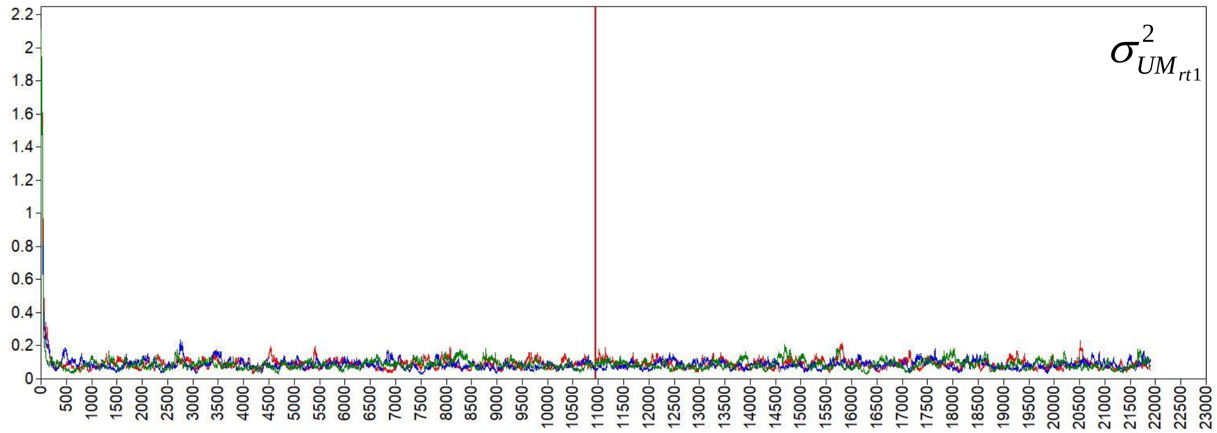
$\sigma^2_{UM_{rt1}}$

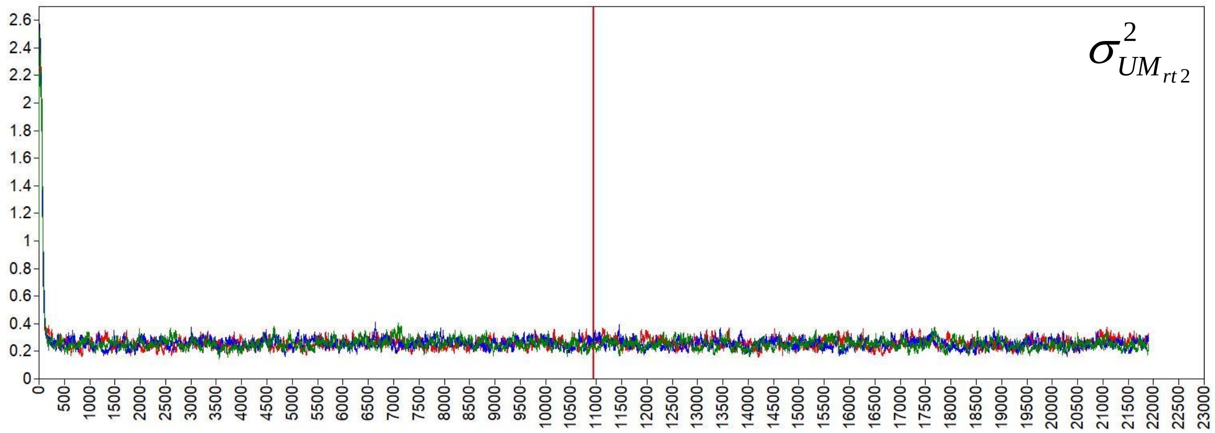
$\sigma^2_{UM_{rt2}}$

DTTL-B

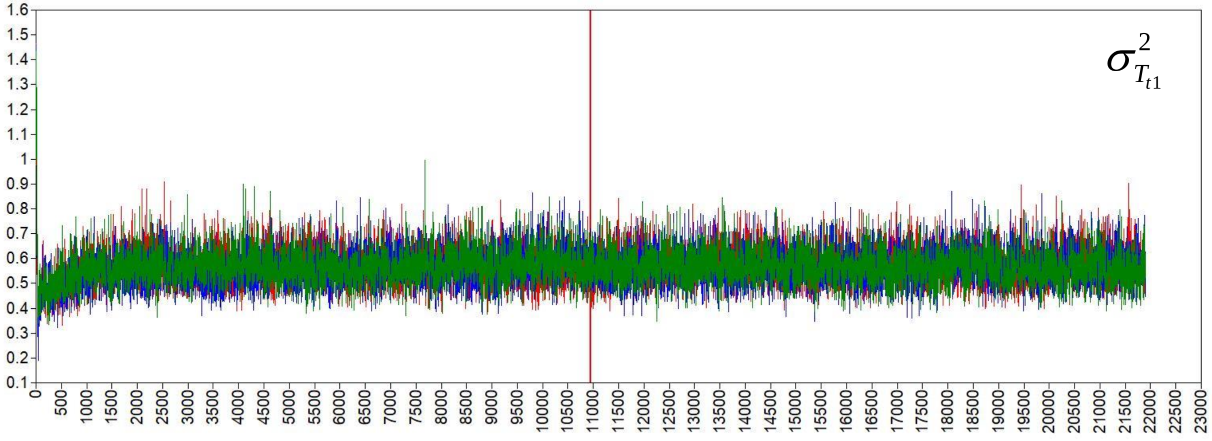

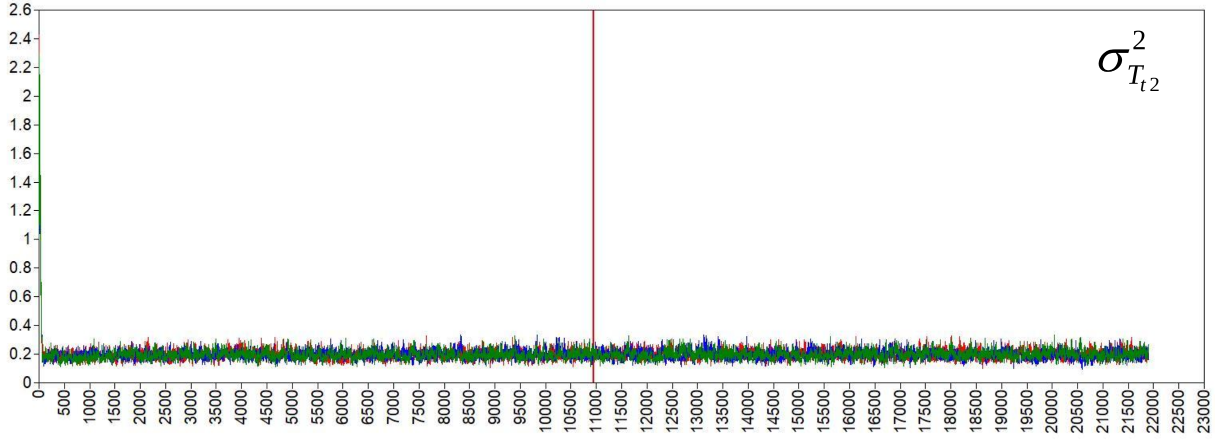

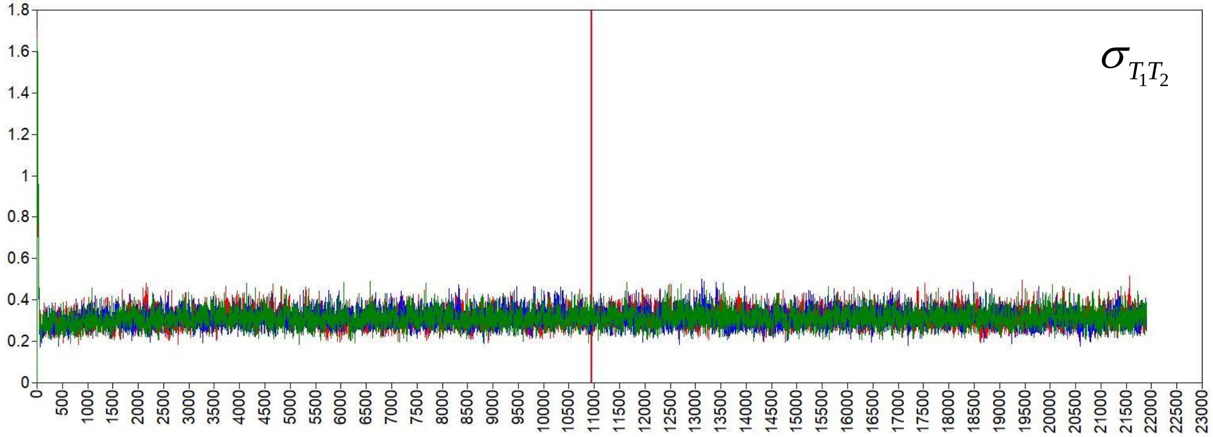

DTTL-B

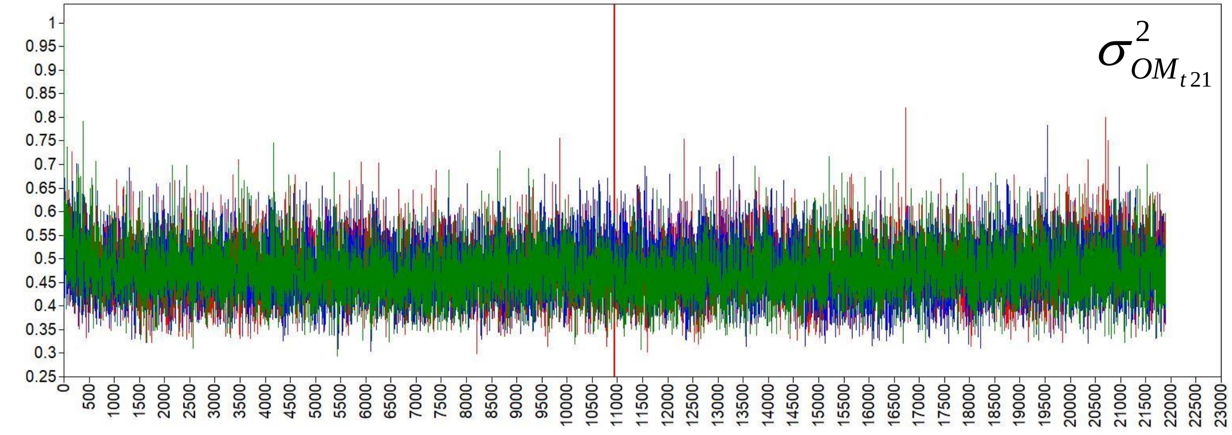

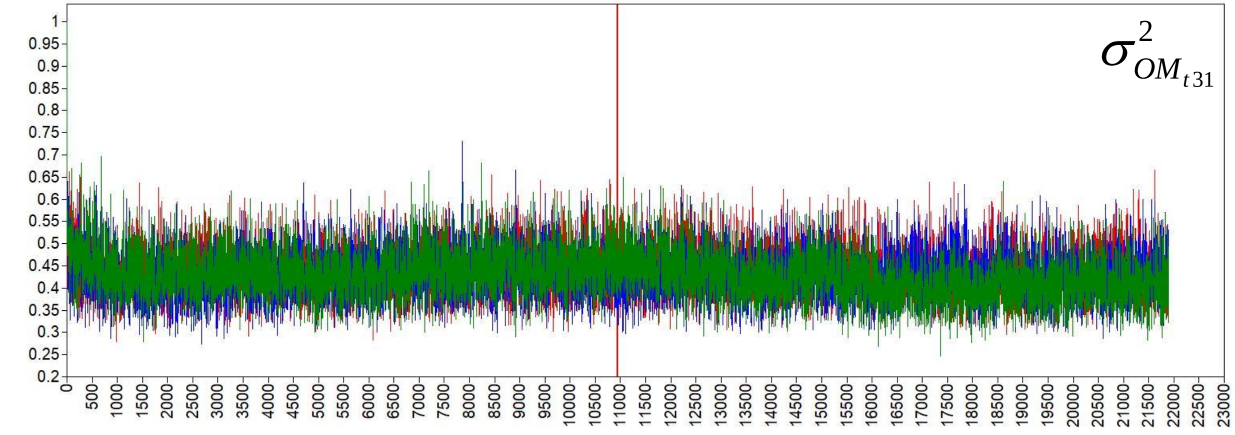

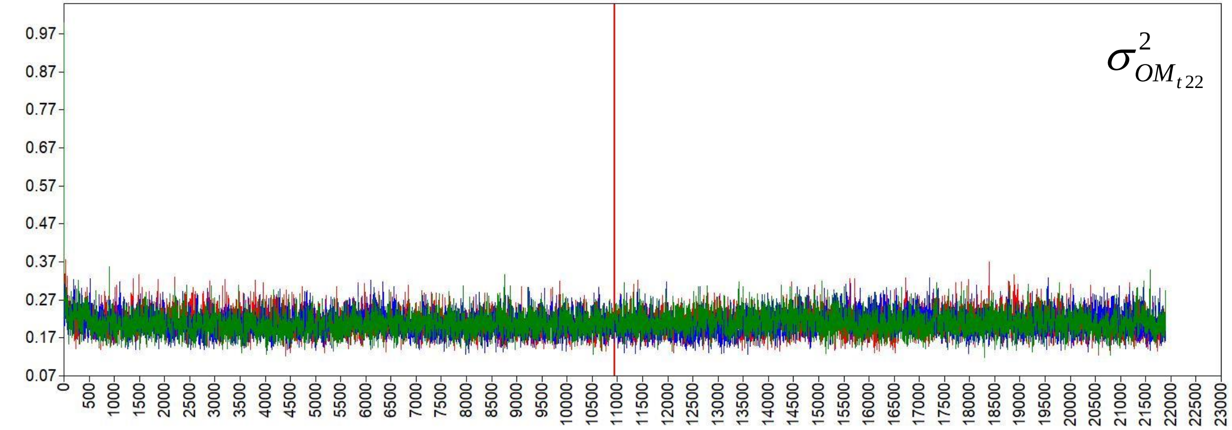

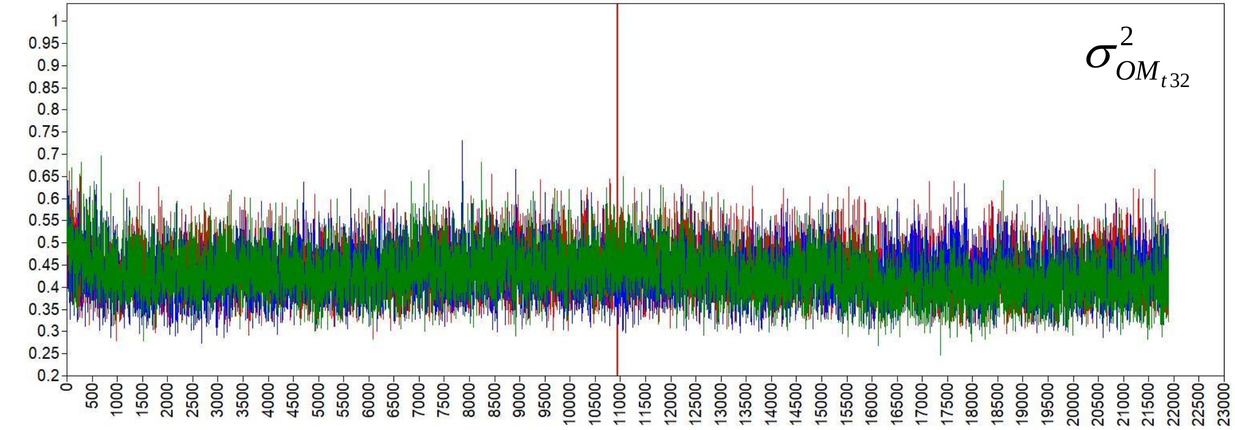

DTTL-B

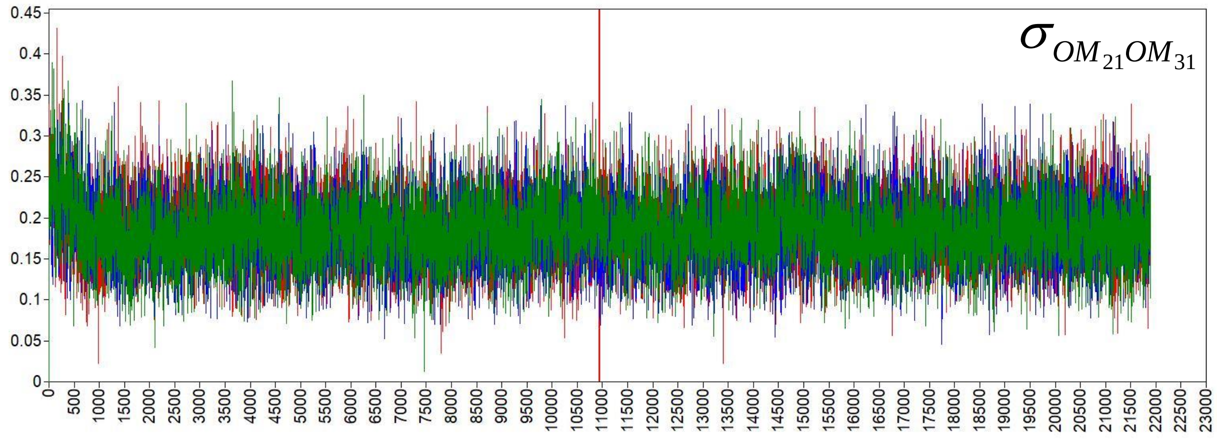
$\sigma_{OM_{21}OM_{31}}$

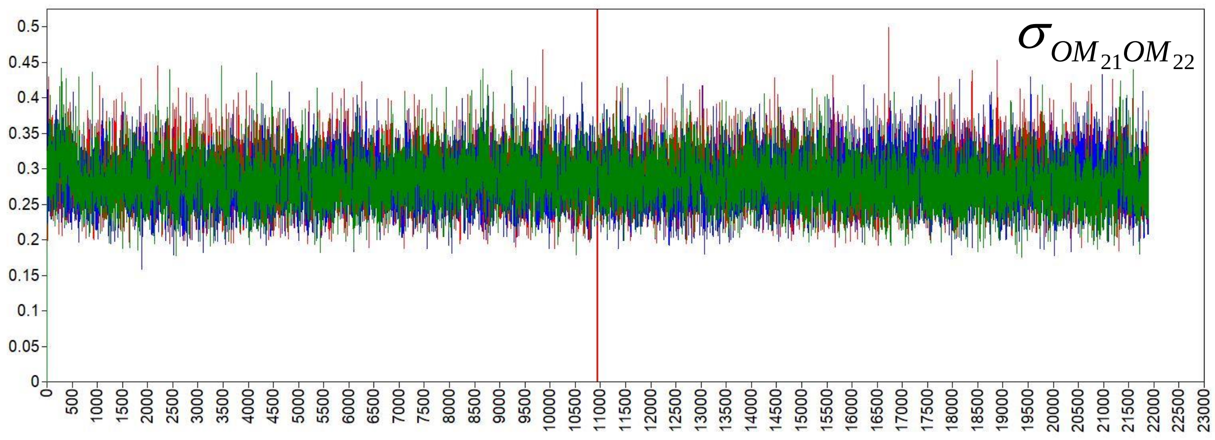
$\sigma_{OM_{21}OM_{22}}$

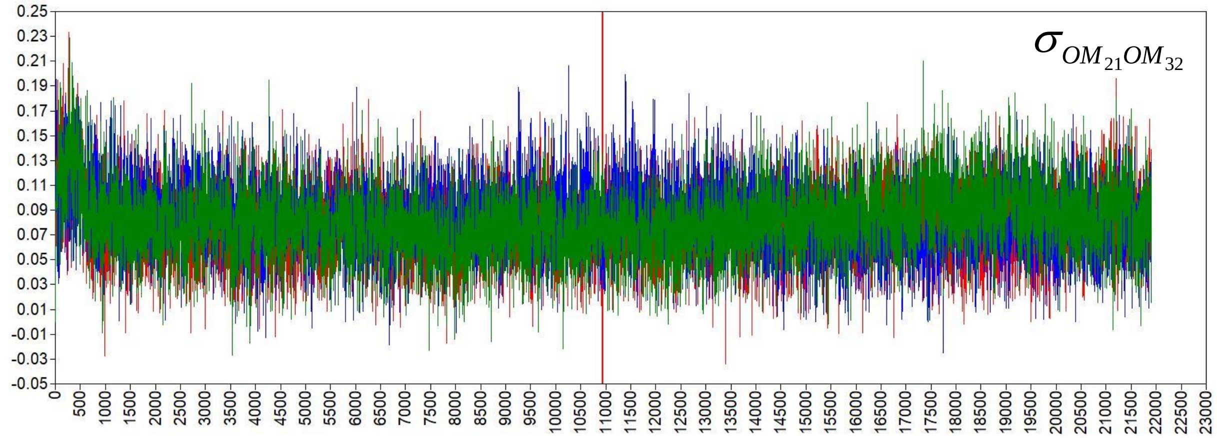
$\sigma_{OM_{21}OM_{32}}$

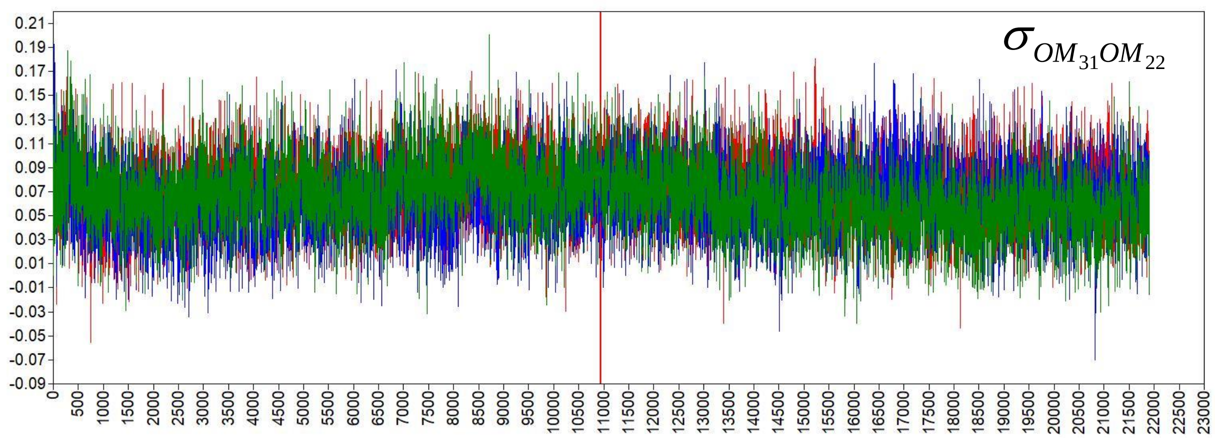
$\sigma_{OM_{31}OM_{22}}$

DTTL-B

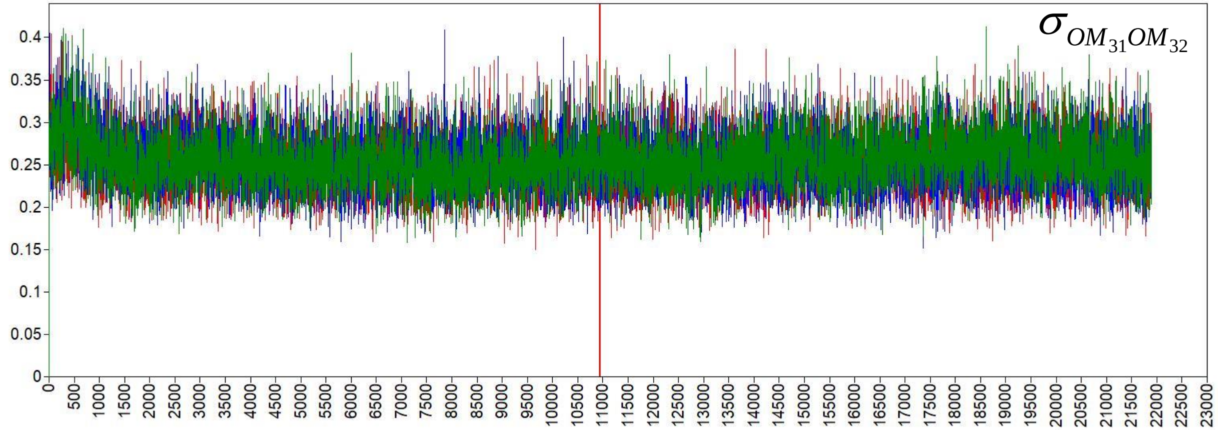

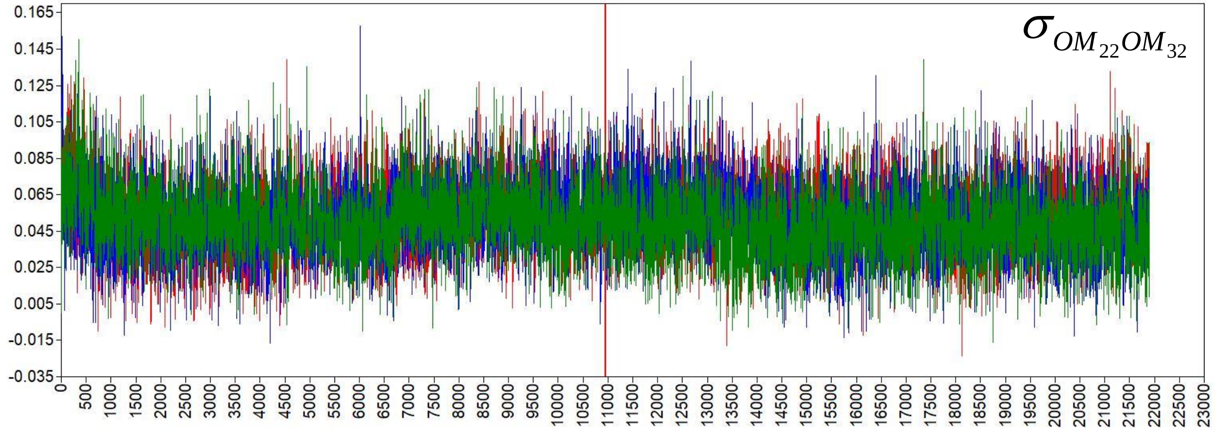

Supplement: Supplementary file 1 [file jintelligence-12-00095-s001.zip › supplementary_material/trace_plots.pdf]
